# Inhibition of fibroblast activation protein ameliorates cartilage matrix degradation and osteoarthritis progression

**DOI:** 10.1038/s41413-022-00243-8

**Published:** 2023-01-02

**Authors:** Aoyuan Fan, Genbin Wu, Jianfang Wang, Laiya Lu, Jingyi Wang, Hanjing Wei, Yuxi Sun, Yanhua Xu, Chunyang Mo, Xiaoying Zhang, Zhiying Pang, Zhangyi Pan, Yiming Wang, Liangyu Lu, Guojian Fu, Mengqiu Ma, Qiaoling Zhu, Dandan Cao, Jiachen Qin, Feng Yin, Rui Yue

**Affiliations:** 1 Department of Joint Surgery, Shanghai East Hospital, Tongji University School of Medicine, Shanghai, 200092 China; 2 Department of Orthopedic Surgery, Shanghai General Hospital, Shanghai Jiao Tong University School of Medicine, Shanghai, 200240 China; 3 Institute for Regenerative Medicine, Shanghai East Hospital, Frontier Science Center for Stem Cell Research, Shanghai Key Laboratory of Signaling and Disease Research, School of Life Sciences and Technology, Tongji University, Shanghai, 200092 China; 4 Department of Cardiology, Shanghai Tenth People’s Hospital, Tongji University School of Medicine, Shanghai, 200072 China; 5 Shanghai Institute of Stem Cell Research and Clinical Translation, Shanghai, 200120 China; 6 Shanghai Clinical Research Center for Aging and Medicine, Shanghai, 200040 China

**Keywords:** Pathogenesis, Bone

## Abstract

Fibroblast activation protein (Fap) is a serine protease that degrades denatured type I collagen, α2-antiplasmin and FGF21. Fap is highly expressed in bone marrow stromal cells and functions as an osteogenic suppressor and can be inhibited by the bone growth factor Osteolectin (Oln). Fap is also expressed in synovial fibroblasts and positively correlated with the severity of rheumatoid arthritis (RA). However, whether Fap plays a critical role in osteoarthritis (OA) remains poorly understood. Here, we found that Fap is significantly elevated in osteoarthritic synovium, while the genetic deletion or pharmacological inhibition of Fap significantly ameliorated posttraumatic OA in mice. Mechanistically, we found that Fap degrades denatured type II collagen (Col II) and Mmp13-cleaved native Col II. Intra-articular injection of rFap significantly accelerated Col II degradation and OA progression. In contrast, Oln is expressed in the superficial layer of articular cartilage and is significantly downregulated in OA. Genetic deletion of Oln significantly exacerbated OA progression, which was partially rescued by Fap deletion or inhibition. Intra-articular injection of rOln significantly ameliorated OA progression. Taken together, these findings identify Fap as a critical pathogenic factor in OA that could be targeted by both synthetic and endogenous inhibitors to ameliorate articular cartilage degradation.

## Introduction

Osteoarthritis (OA) is one of the most common orthopedic diseases worldwide,^1,2^ with an approximately 26.6% prevalence rate among people over 45 years old.^3^ The most prominent pathological changes of OA include articular cartilage degeneration, osteophyte formation, low-grade inflammation and subchondral bone remodeling.^4–8^ After initial mechanical erosion during aging, articular cartilage and synovium express a panel of proteolytic enzymes and proinflammatory factors that accelerate cartilage matrix degradation and OA progression.^9–11^ Early-stage OA patients can be treated by microfracture, osteochondral allograft transplantation, or biomaterial implantation,^12,13^ while late-stage OA patients can only be treated by replacement plastic surgeries.^14^ Pharmacological treatments such as nonsteroidal anti-inflammatory drugs and intra-articular glucocorticoid injection are recommended by most guidelines to relieve inflammation and pain.^15–17^ In contrast, the effectiveness of other drugs, such as glucosamine, chondroitin and hyaluronic acid, remains controversial.^15–17^ Therefore, drugs with higher efficacy for OA treatment are under intensive investigation.^18^

Aggrecan (Acan) and type II collagen (Col II) are the two major components of the cartilage matrix.^19^ Whereas Acan degradation is mainly mediated by a disintegrin and metalloprotease with thrombospondin motifs (ADAMTS) family proteases,^20^ Col II degradation is mainly catalyzed by matrix metalloproteinases (MMPs).^21^ Collagenases, such as MMP1/8/13, are responsible for unwinding and cleaving the triple-helical Col II fiber into large fragments,^22–25^ after which gelatinases, such as MMP2/9, further digest them into smaller peptides.^26,27^ Although MMPs were initially considered potential drug targets for OA treatment, none of the nonselective MMP inhibitors passed clinical trials due to potential side effects, including joint stiffness, inflammation and pain.^28,29^ In contrast, selective MMP inhibitors demonstrated low solubility and permeability,^23^ precluding their clinical applications. In addition to ADAMTS and MMPs, serine proteases, such as thrombin, matriptase and HtrA1, were also implicated in OA progression.^30–33^ However, no serine protease inhibitors have been approved to treat OA.

Fibroblast activation protein (Fap) is a serine protease with both dipeptidyl peptidase and endopeptidase activities.^34,35^ It is known to degrade denatured type I collagen (Col I), alpha2-antiplasmin and FGF21 in vivo^35–39^ as well as several other substrates in vitro.^40,41^ Fap was upregulated in activated fibroblasts and critically involved in tumor microenvironment formation, inflammation and wound healing.^42–47^ In the musculoskeletal system, *Fap* is highly expressed in bone marrow stromal cells (BMSCs) and osteoblasts, which function as osteogenic suppressors.^48^ Genetic deletion or pharmacological inhibition of Fap promotes bone formation and inhibits bone resorption, suggesting that it is a potential anti-osteoporosis drug target.^48^ Interestingly, Fap was also detected in the synovium of OA patients, although at a lower level compared to rheumatoid arthritis (RA) patients.^49,50^ Whether Fap plays an important role in regulating OA progression remains poorly understood.

Osteolectin (Oln), also known as Clec11a or Scgf, was initially identified as a growth factor that promotes hematopoietic colony formation in vitro.^51,52^ Our previous in vivo studies showed that *Oln* is highly expressed in osteochondral lineage cells and functions as an anabolic factor that promotes bone formation by activating the Wnt pathway.^53,54^ We also demonstrated that Oln interacts with Fap and inhibits its protease activity,^48^ suggesting a novel mechanism by which Oln promotes bone formation and osteogenic differentiation.^48^ In the present study, we found that synovium-derived Fap exacerbates OA progression. Oln forms a protective barrier on the cartilage surface and shows the opposite effects. A mechanistic study revealed that Fap promotes the degradation of Col II in osteoarthritic cartilage, which could be ameliorated by intra-articular injection of a small molecule inhibitor of Fap or recombinant Oln.

## Results

### Fap is significantly upregulated in OA synovium

**Fig. 1:**
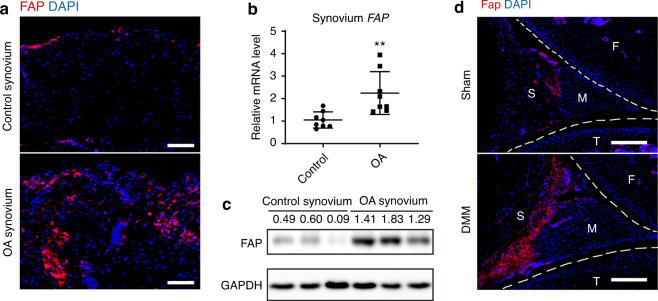
Expression analysis of Fap in OA synovium. **a** Immunostaining of human FAP in the synovium of control and OA patients. DAPI staining indicates the nucleus. Scale bars: 100 μm. **b** qPCR analysis of human *FAP* mRNA levels in the synovium of control and OA patients (*n* = 8 samples per group). **c** Western blot analysis of human FAP protein levels in the synovium of control and OA patients (*n* = 3 samples per group). **d** Immunostaining of mouse Fap in the knee joint of sham and DMM-treated mice. Sham or DMM surgery was performed in 8-week-old Fap^LacZ/+^ mice, which were sacrificed 8 weeks later, and LacZ immunostaining was used to detect Fap expression at the posterior horn of the medial meniscus (F: femur; T: tibia; M: meniscus; S: synovium). DAPI staining indicates the nucleus. Scale bars: 100 μm. The statistical significance was assessed using two-tailed Student’s unpaired *t* tests. Data are presented as the mean ± SD (**P < 0.01)

To test whether Fap is involved in OA pathogenesis, we first analyzed its expression in the synovium of patients diagnosed with OA or acute joint injury (control). Immunofluorescence staining showed that human FAP was mainly detected in the OA synovium (Fig. 1a and Supplementary Fig. 1a). Quantitative real-time PCR (qPCR) confirmed that *FAP* mRNA was significantly upregulated in OA synovium compared to control synovium (Fig. 1b). Western blot analysis showed an even greater increase in FAP protein levels in the OA synovium (Fig. 1c). Consistent with the human data, Fap was only marginally detected in the synovium of sham-operated mice, whereas it was significantly upregulated in OA synovium after surgical destabilization of the medial meniscus (DMM) (Fig. 1d and Supplementary Fig. 1b). To test whether the inflammatory microenvironment induced by OA^10^ regulates Fap expression, we stimulated primary human synovial fibroblasts with IL-1β ex vivo. We found that *FAP* mRNA was significantly increased in a time-dependent manner, suggesting that proinflammatory factors promote Fap expression in the synovium (Fig. S1c).

### Genetic deletion or pharmacological inhibition of Fap ameliorates OA progression

To test whether Fap regulates OA progression, we performed unilateral knee joint DMM surgery in *Fap*-deficient (Fap KO) and wild-type control mice. Three days after the surgery, we administered weekly intra-articular injections of a Fap-selective small molecule inhibitor (FAPi, 40 μg·kg^−1^ body weight)^55^ or vehicle control (PBS) in the DMM-treated knee joints for 8 weeks. Mice were then sacrificed, and knee joint sections of the medial compartment were stained with safranin O and fast green.

**Fig. 2:**
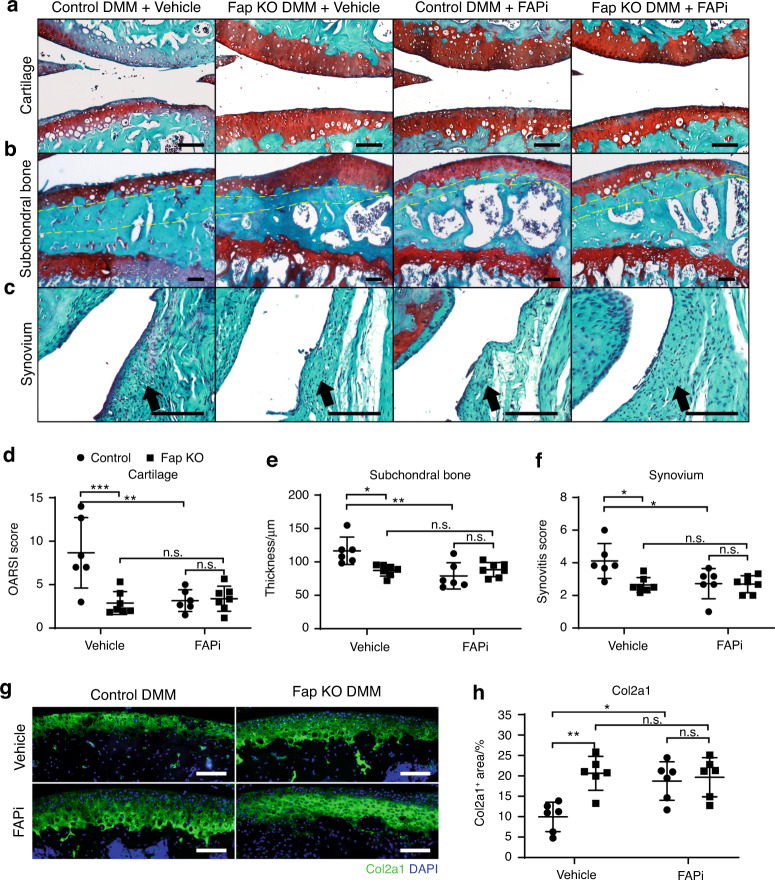
Fap promotes OA progression in a mouse DMM model. **a**–**c** Safranin O/Fast Green staining in control and Fap KO mice treated with FAPi or vehicle after DMM surgery. Weekly intra-articular administration of FAPi (40 μg/kg body weight) or vehicle (PBS) in 10-week-old mice was started 3 days after the surgery and continued for 8 weeks before paraffin sectioning and safranin O/fast green staining of the knee joints. Representative cartilage erosion (**a**, top: femur, bottom: tibia), subchondral bone thickening (**b**) and synovitis (**c**) images are shown (*n* = 6–7 mice per genotype in each treatment group). Yellow dotted lines indicate the subchondral bone plate. Arrows indicate the synovium. Scale bars: 100 μm. **d**–**f** Quantification of the OARSI score (**d**), subchondral bone thickness (**e**) and synovitis score (**f**). OARSI scores were calculated based on the erosion of the medial tibial plateau cartilage (**a**, bottom). Subchondral bone thickness was measured as the mean distance of five evenly distributed measuring points between the lower edge of the articular cartilage and the roof of the cancellous bone. Synovitis scores were calculated by summing the enlargement of the synovial lining cell layer, density of the resident cells and inflammatory infiltration. **g**, **h** Immunostaining of Col II in the knee joints (**g**) with quantifications (**h**) (*n* = 6 mice per genotype in each treatment group). DAPI staining indicates the nucleus. Scale bars: 100 μm. The statistical significance was assessed using two-way ANOVAs with Sidak’s multiple comparison tests. Data are presented as the mean ± SD (*P < 0.05, **P < 0.01, ***P < 0.001)

Histomorphometry analyses in the contralateral knee joint without DMM surgery showed no significant differences between Fap KO and control mice (Fig. S2a–f), suggesting that Fap is dispensable for knee joint development and maintenance under a steady state. In contrast, the knee joint with DMM surgery showed significant cartilage erosion, subchondral bone thickening and synovitis in control mice (Fig. 2a–f). Notably, these OA symptoms were significantly ameliorated in Fap KO mice (Fig. 2a–f). Intra-articular administration of FAPi in control mice also significantly ameliorated cartilage erosion, subchondral bone thickening and synovitis compared to the vehicle group (Fig. 2a–f). Importantly, FAPi administration showed no therapeutic effects in Fap KO mice, indicating that the inhibitor functioned in a Fap-dependent manner (Fig. 2a–f). Immunofluorescent staining of Col2a1 in the articular cartilage showed similar results (Fig. 2g, h). We also used micro-CT to analyze osteophyte formation and found that the intra-articular administration of FAPi significantly ameliorated osteophyte formation in control but not Fap KO mice (Fig. S3a, b). There was also a trend toward decreased osteophyte formation in Fap KO mice compared to control mice after vehicle treatment (Supplementary Fig. 3a, b).

**Fig. 3:**
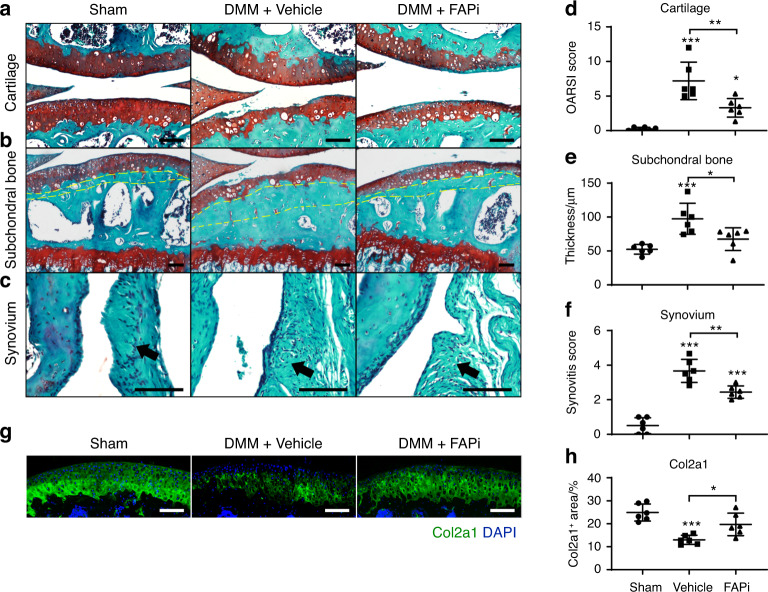
Pharmacological inhibition of Fap ameliorates joint symptoms after the onset of OA. **a**–**c** Safranin O/Fast Green staining in wild-type mice treated with FAPi or vehicle after DMM surgery. Weekly intra-articular administration of FAPi (40 μg·kg^−1^ body weight) or vehicle (PBS) was performed 4 weeks after DMM surgery in 10-week-old mice and continued for 8 weeks before paraffin sectioning and safranin O/fast green staining of the knee joints. Representative cartilage erosion (**a**, top: femur, bottom: tibia), subchondral bone thickening (**b**) and synovitis (**c**) images are shown (*n* = 6 mice per group). Yellow dotted lines indicate the subchondral bone plate. Arrows indicate the synovium. Scale bars: 100 μm. **d**–**f** Quantification of the OARSI score (**d**), subchondral bone thickness (**e**) and synovitis score (**f**). **g**, **h** Immunostaining of Col II in the knee joints (**g**) with quantification (**h**) (*n* = 6 mice per group). DAPI staining indicates the nucleus. Scale bars: 100 μm. The statistical significance was assessed using one-way ANOVAs with Tukey’s multiple comparison tests. Data are presented as the mean ± SD (*P < 0.05, **P < 0.01, ***P < 0.001)

To test whether FAPi administration could ameliorate articular symptoms after the onset of OA, we first performed DMM surgery in wild-type mice and waited for 4 weeks. We then administered weekly intra-articular injections of FAPi or vehicle control for another 8 weeks. Compared to sham-operated mice, vehicle-treated mice showed profound cartilage erosion, subchondral bone thickening and synovitis after DMM (Fig. 3a–f), which were significantly ameliorated by FAPi treatment (Fig. 3a–f). Immunofluorescent staining of Col2a1 in the articular cartilage showed similar results (Fig. 3g, h). Taken together, these data showed that the genetic deletion or pharmacological inhibition of Fap significantly ameliorated OA progression.

### Fap does not regulate chondrocyte function in a cell-autonomous manner

Fap is expressed in perichondral mesenchymal cells surrounding the cartilage primordia during mouse embryonic development.^56^ Consistent with this, Fap was mainly detected in the synovium, but not articular cartilage, of normal and OA knee joints in adult mice (Fig. 1d). To test whether Fap regulates chondrocyte function in a cell-autonomous manner, we cultured primary articular chondrocytes from Fap KO or wild-type newborn mice and stimulated the cells with IL-1β to mimic OA pathogenesis.^57–59^ Consistent with a previous study,^60^ qPCR analysis in control chondrocytes showed significantly decreased *Acan* and *Col2a1* mRNA levels and significantly increased *Mmp3* mRNA levels after IL-1β stimulation (Fig. S4a–c). Compared to control chondrocytes, Fap KO chondrocytes showed similar *Acan*, *Col2a1* and *Mmp3* expression before and after IL-1β stimulation (Fig. S4a–c). Consistent with this, western blot analysis showed significantly decreased Acan and Col2a1 protein levels and significantly increased Mmp3 protein levels after IL-1β stimulation (Fig. S4d, e), with no significant differences between Fap KO and control chondrocytes (Supplementary Fig. 4d, e).

Next, we treated primary wild-type chondrocytes with 10 μg/ml FAPi for different periods ex vivo but still found no significant changes in *Acan*, *Col2a1* or *Mmp3* mRNA levels (Supplementary Fig. 5a–c). Different doses of FAPi were also administered with or without IL-1β stimulation during ex vivo culture, which did not significantly affect the expression of *Acan*, *Col2a1* or *Mmp3* in primary wild-type chondrocytes (Fig. S5d–f). Taken together, these data suggest that Fap does not autonomously regulate chondrocyte function.

### Col II is a novel substrate of Fap both in vitro and in vivo

**Fig. 4:**
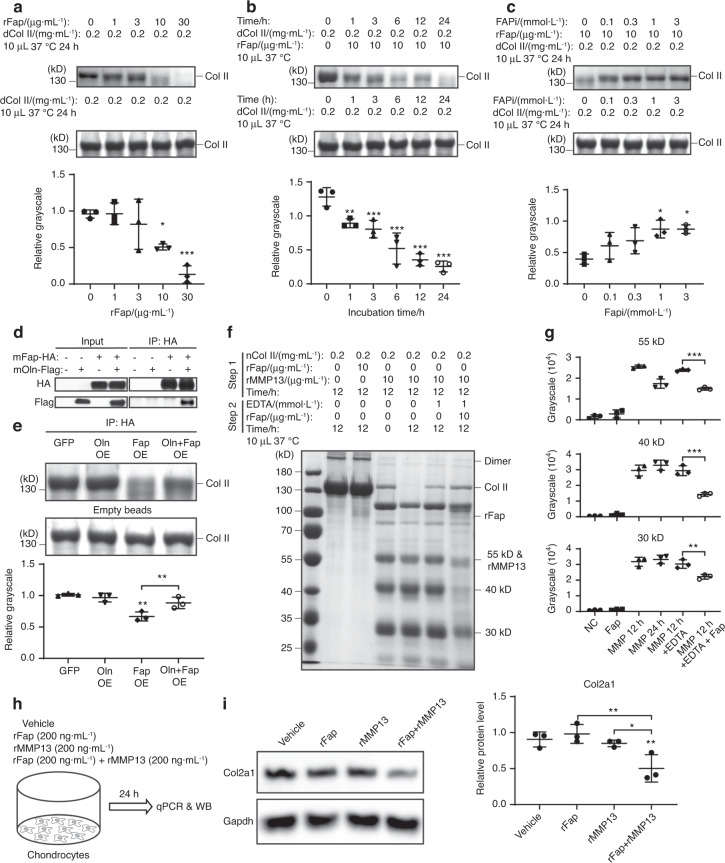
Fap degrades denatured or MMP13-cleaved Col II. **a** Fap degrades denatured Col II in a dose-dependent manner. Denatured Col II (boiled at 95 °C for 10 min) was incubated with different amounts of rFap at 37 °C for 24 h. Samples were separated by SDS‒PAGE and quantified by colloidal blue staining (*n* = 3 independent experiments). **b** Fap degrades denatured Col II in a time-dependent manner. Denatured Col II was incubated with rFap at 37 °C for 0–24 h (*n* = 3 independent experiments). **c** FAPi inhibits Fap-mediated degradation of denatured Col II. Different doses of FAPi were preincubated with rFap for 30 min before denatured Col II was added and further incubated at 37 °C for 24 h (*n* = 3 independent experiments). **d** Immunoprecipitation of Fap. HEK293T cells were transfected with GFP control, Oln-Flag, Fap-HA, or Oln-Flag + Fap-HA. Two days after transfection, Fap was immunoprecipitated from total cell lysates with 10 μL anti-HA affinity gel. Five percent total cell lysates were loaded as an input control (*n* = 3 independent experiments). **e** Oln inhibits the Fap-mediated degradation of denatured Col II. Col II was coincubated with immunoprecipitated samples (in 10 μL anti-HA affinity gel) at 37 °C for 24 h (*n* = 3 independent experiments). **f** Fap degrades MMP13-cleaved Col II. Native Col II was preincubated with rFap or rMMP13 at 37 °C for 12 h. EDTA was then added to the reaction mixture with or without rFap and incubated at 37 °C for another 12 h (*n* = 3 independent experiments). **g** Grayscale quantification of the 55, 40 and 30 kDa digestion bands in (**f**). **h** Experimental design. Primary chondrocytes were stimulated with vehicle control (PBS), 200 ng·mL^−1^ rFap, 200 ng·mL^−1^ rMMP13, or 200 ng·mL^−1^ rFap plus 200 ng·mL^−1^ rMMP13 for 24 h. **i** Western blot analysis of mouse Col2a1 protein levels in primary chondrocytes stimulated as in (**h**) (*n* = 3 independent experiments). The statistical significance was assessed using one-way ANOVAs with Tukey’s multiple comparison tests. Data are presented as the mean ± SD (*P < 0.05, **P < 0.01, ***P < 0.001)

Fap participates in extracellular matrix remodeling in many diseases, such as pancreatic cancer, myocardial infarction, and thin-cap fibroatheroma,^61–63^ suggesting that it might play similar roles during OA progression. Since Fap is a serine protease that degrades denatured or Mmp1-cleaved Col I,^35,63–65^ we hypothesized that Fap could also degrade Col II, the key component of the cartilage matrix. Similar to native Col I, native Col II was resistant to digestion by recombinant Fap (rFap) (Supplementary Fig. 6a). Thus, we heat-inactivated Col II at 95 °C for 10 min to depolymerize it into monomers and then incubated it with rFap at 37 °C for 24 h. Remarkably, we observed a dose- and time-dependent degradation of denatured Col II by rFap (Fig. 4a, b). Preincubation with FAPi for 30 min dose-dependently inhibited the degradation of Col II by rFap (Fig. 4c), further validating the efficacy of FAPi. Our recent study demonstrated that Oln functions as an endogenous Fap inhibitor to promote bone formation.^48^ To test whether Oln inhibits the degradation of denatured Col II by rFap, we overexpressed mouse Fap, alone or together with mouse Oln, in HEK293T cells and immunoprecipitated Fap (Fig. 4d). Consistent with our previous study,^48^ Oln physically interacts with Fap, which does not affect the amount of Fap that was expressed or precipitated (Fig. 4d). Immunoprecipitated Fap also degraded denatured Col II, which could be partially inhibited by Oln coexpression (Fig. 4e).

Next, we tested whether Fap exhibits gelatinase activity similar to MMP2/9, which further digests MMP13-cleaved Col II into smaller fragments.^26,27^ Recombinant human MMP13 (rMMP13, 10 μg·mL^−1^) was able to digest native Col II into 6 major fragments in a time-dependent manner (Fig. 4f). When we terminated the protease activity of MMP13 using EDTA after 12 h of incubation and then added rFap (10 μg·mL^−1^) for another 12 h, the 55, 40 and 30 kD fragments could be further digested by rFap (Fig. 4f, g). To test whether Fap degrades Col II in a pathological setting, we measured the Fap level in synovial fluids from OA patients by ELISA and found that its average concentration was approximately 150 ng·mL^−1^ (Fig. S6b). A previous study reported that the concentration of MMP13 in OA synovial fluids is approximately 200 ng·mL^−1^.^66,67^ We then cultured primary chondrocytes from newborn mice and administered rFap (200 ng·mL^−1^), rMMP13 (200 ng·mL^−1^), or rFap plus rMMP13 for 24 h (Fig. 4h). qPCR analysis showed no significant changes in *Col2a1* mRNA levels in any treatment group (Supplementary Fig. 6c). Western blot analysis showed unaltered Col2a1 protein levels in total cell lysates after rFap or rMMP13 treatment (Fig. 4i) but significantly decreased Col2a1 protein levels in the rFap plus rMMP13 group (Fig. 4i). Together, these data indicate that Fap works in concert with MMP13 to degrade Col II both in vitro and ex vivo.

**Fig. 5:**
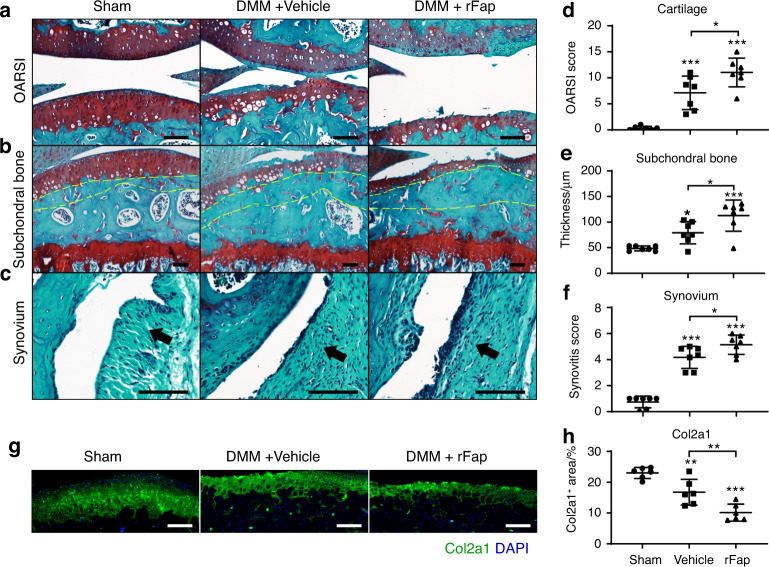
rFap accelerates joint symptoms after the onset of OA. **a**–**c** Safranin O/Fast Green staining in wild-type mice administered rFap or vehicle after DMM surgery. Weekly intra-articular administration of rFap (40 μg/kg body weight) or vehicle (PBS) was performed 4 weeks after DMM surgery in 10-week-old mice and continued for 8 weeks before paraffin sectioning and safranin O/fast green staining of the knee joints. Representative cartilage erosion (**a**, top: femur, bottom: tibia), subchondral bone thickening (**b**) and synovitis (**c**) images are shown (*n* = 7 mice per group). Yellow dotted lines indicate the subchondral bone plate. Arrows indicate the synovium. Scale bars: 100 μm. **d**–**f** Quantification of the OARSI score (**d**), subchondral bone thickness (**e**) and synovitis score (**f**). **g**, **h** Immunostaining of Col II in the knee joints (**g**) with quantification (**h**) (*n* = 6 mice per group). DAPI staining indicates the nucleus. Scale bars: 100 μm. The statistical significance was assessed using one-way ANOVAs with Tukey’s multiple comparison tests. Data are presented as the mean ± SD (*P < 0.05, **P < 0.01, ***P < 0.001)

To test whether rFap exacerbates OA progression and Col II degradation in vivo, we administered weekly intra-articular injections of rFap after the onset of OA (4 weeks after DMM surgery) for 8 weeks. Compared to vehicle controls, rFap-treated mice showed significantly increased articular cartilage erosion, subchondral bone thickening and synovitis (Fig. 5a–f). Micro-CT analysis showed increased osteophyte formation after rFap injection (Fig. S7a, b). Immunofluorescent staining of Col2a1 showed a significant decrease in the Col2a1^+^ area in the articular cartilage of rFap-treated mice (Fig. 5g, h). Together with the fact that the Col II level was significantly elevated in the articular cartilage by genetic or pharmacological inhibition of Fap after DMM (Figs. 2g, h and 3g, h), we concluded that Col II is a novel substrate of Fap in vivo.

To test whether Fap degrades Acan, another key component of the cartilage matrix, we incubated rFap with recombinant human Acan (G1-IGD-G2 domain) at 37 °C for 24 h. rFap could not digest either native or denatured Acan (Supplementary Fig. 6d, e), indicating that Fap specifically degrades Col II in the cartilage matrix.

### Oln is significantly downregulated in OA cartilage

**Fig. 6:**
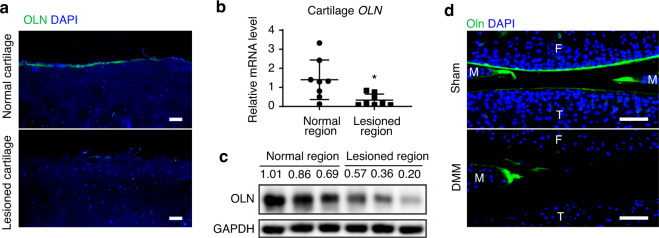
Expression analysis of Oln in OA cartilage. **a** Immunostaining of human OLN in the articular cartilage of control and OA patients. DAPI staining indicates the nucleus. Scale bars: 100 μm. **b** qPCR analysis of *OLN* mRNA levels in the articular cartilage of OA patients. Cartilage samples were separated into normal and lesioned regions based on their morphology (*n* = 8 samples per group). **c** Western blot analysis of OLN protein levels in the articular cartilage from OA patients (*n* = 3 samples per group). **d** Immunostaining of mouse Oln in the knee joints of sham and DMM-treated mice. Sham or DMM surgery was performed in 8-week-old wild-type mice, which were analyzed 8 weeks later (F: Femur; T: Tibia). DAPI staining indicates the nucleus. Scale bars: 100 μm. The statistical significance was assessed using two-tailed Student’s unpaired *t* tests. Data are presented as the mean ± SD (*P < 0.05)

Since Oln functions as an endogenous inhibitor of Fap,^48^ we tested whether Oln also regulates OA progression. Our previous study showed that Oln is highly expressed in growth plate chondrocytes, osteoblasts and BMSCs.^53^ Immunofluorescent staining showed that Oln is also expressed in the superficial layer of normal human articular cartilage but absent in the lesioned cartilage from OA patients (Fig. 6a and Supplementary Fig. 8a). qPCR analysis showed that *OLN* levels were significantly decreased in lesioned OA cartilage compared to adjacent normal cartilage (Fig. 6b), which was confirmed by western blotting (Fig. 6c). In contrast to Fap, Oln was not detected in either normal or OA patient synovium (Supplementary Fig. 8b).

We also analyzed the localization of Oln in the mouse knee joint and found that it is expressed in the superficial layer of articular cartilage and meniscus (Fig. 6d). Consistent with the human data, mouse Oln was dramatically downregulated after DMM surgery (Fig. 6d). Mouse Oln was also marginally detected in the boundary between the synovium and meniscus, which was not affected by DMM (Supplementary Fig. 8c). No Oln signal could be detected in IgG control or *Oln*-deficient (Oln KO) mice (Supplementary Fig. 8d). Interestingly, *Oln* mRNA was significantly decreased in primary chondrocytes after IL-1β stimulation ex vivo (Supplementary Fig. 8e), suggesting that proinflammatory factors inhibit *Oln* expression during OA progression.

### Genetic deletion of *Oln* exacerbates OA progression

**Fig. 7:**
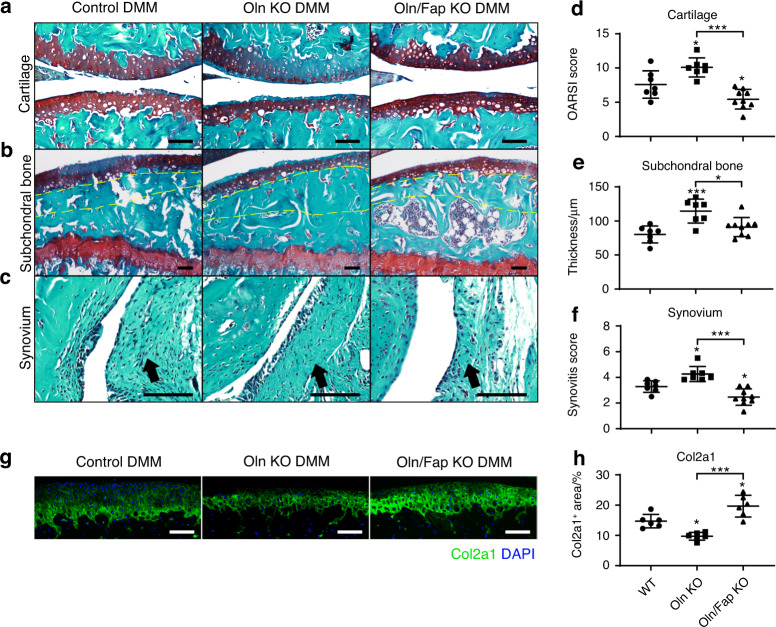
Oln inhibits OA progression in a Fap-dependent manner. **a**–**c** Safranin O/Fast Green staining in control, Oln KO and Oln/Fap double KO mouse knee joints 8 weeks after DMM surgery. DMM surgery was performed in 10-week-old mice. Representative cartilage erosion (**a**, top: femur, bottom: tibia), subchondral bone thickening (**b**) and synovitis (**c**) images are shown (*n* = 7–9 mice per genotype). Yellow dotted lines indicate the subchondral bone plate. Arrows indicate the synovium. Scale bars: 100 μm. **d**–**f** Quantification of the OARSI score (**d**), subchondral bone thickness (**e**) and synovitis score (**f**). **g**, **h** Immunostaining of Col II in the knee joints (**g**) with quantification (**h**) (*n* = 6 mice per genotype). DAPI staining indicates the nucleus. Scale bars: 100 μm. The statistical significance was assessed using one-way ANOVAs with Tukey’s multiple comparison tests. Data are presented as the mean ± SD (*P < 0.05, **P < 0.01, ***P < 0.001)

To test whether Oln regulates OA progression, we performed DMM surgery in Oln KO, Oln/Fap KO (double KO) and wild-type control mice. Histomorphometry analysis showed no significant differences among Oln KO, double KO and control mice in the contralateral knee joints without DMM surgery (Fig. S9a–f), suggesting that neither Oln nor Fap are required for articular cartilage development or maintenance under steady state. In the knee joints with DMM surgery, Oln KO mice showed significantly increased cartilage erosion, subchondral bone thickening, and synovitis compared to control mice (Fig. 7a–f). Importantly, these defects were significantly ameliorated in double KO mice (Fig. 7a–f). Col2a1 staining of the articular cartilage showed similar results (Fig. 7g, h).

Consistent with this finding, weekly intra-articular administration of FAPi also significantly ameliorated OA progression in Oln KO mice (Fig. S10a–h). Oln deficiency did not affect Acan and Col2a1 levels in primary chondrocytes after IL-1β stimulation, while there was a modest but significant increase in Mmp3 levels compared to those in wild-type controls (Fig. S11a–e). Taken together, these findings indicate that the genetic deletion of *Oln* exacerbates OA progression, which could be partially rescued by Fap deletion or inhibition.

### Intra-articular injection of recombinant Oln attenuates OA progression

To test whether recombinant Oln (rOln) showed similar therapeutic effects as those of FAPi, we administered weekly intra-articular injections of recombinant rOln (240 μg·kg^−1^ body weight) or vehicle control (0.05 mg·mL^−1^ hyaluronic acid) in wild-type mice 3 days after DMM surgery for a total of 8 weeks. Histomorphometry analysis showed significantly decreased cartilage erosion, subchondral bone thickening, and synovitis compared to vehicle controls (Fig. S12a–f), which was confirmed by Col2a1 staining of articular cartilage (Fig. S12g, h).

**Fig. 8:**
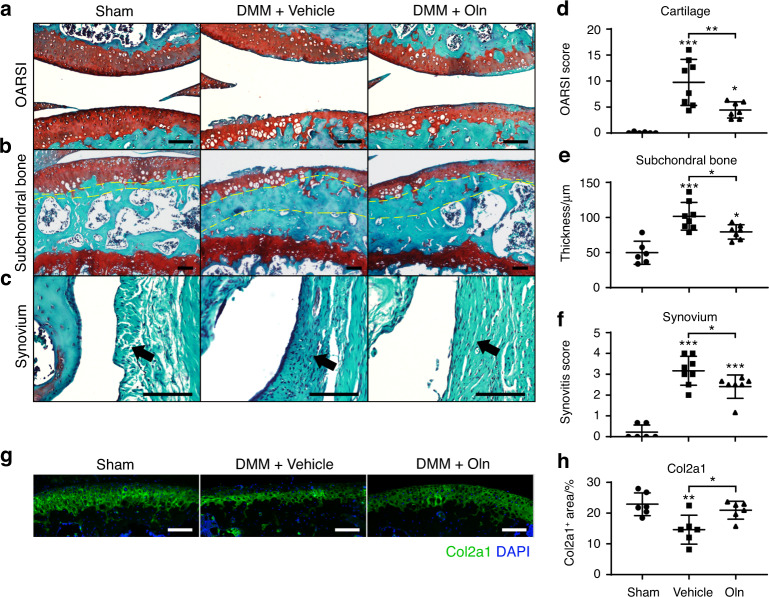
rOln ameliorates joint symptoms after the onset of OA. **a**–**c** Safranin O/Fast Green staining in wild-type mice treated with rOln or vehicle after DMM surgery. Weekly intra-articular administration of rOln (240 μg·kg^−1^ body weight) or vehicle (0.05 mg·mL^−1^ hyaluronic acid) was performed 4 weeks after DMM surgery in 10-week-old mice and continued for 8 weeks before paraffin sectioning and safranin O/fast green staining of the knee joints. Representative cartilage erosion (**a**, top: femur, bottom: tibia), subchondral bone thickening (**b**) and synovitis (**c**) images are shown (*n* = 6–8 mice per treatment). Yellow dotted lines indicate the subchondral bone plate. Arrows indicate the synovium. Scale bars: 100 μm. **d**–**f** Quantification of the OARSI score (**d**), subchondral bone thickness (**e**) and synovitis score (**f**). **g**, **h** Immunostaining of Col II in the knee joints (**g**) with quantification (**h**) (*n* = 6 mice per treatment). DAPI staining indicates the nucleus. Scale bars: 100 μm. The statistical significance was assessed using one-way ANOVAs with Tukey’s multiple comparison tests. Data are presented as the mean ± SD (*P < 0.05, **P < 0.01, ***P < 0.001)

To test whether rOln could ameliorate articular symptoms after the onset of OA, we administered weekly intra-articular injections of rOln or vehicle control in wild-type mice 4 weeks after DMM surgery for a total of 8 weeks. The vehicle-treated DMM group showed profound cartilage erosion, subchondral bone thickening and synovitis compared to the sham-operated group, and these effects were significantly ameliorated in the rOln-treated group (Fig. 8a–f). Similar results were obtained by immunostaining of Col2a1 in articular cartilage (Fig. 8g, h). Taken together, these data showed that intra-articular rOln administration significantly attenuated OA progression.

## Discussion

OA is a degenerative orthopedic disease that severely affects the quality of life of elderly people worldwide. Apart from surgical treatments, very few medicines have been developed to effectively treat OA symptoms by either promoting cartilage regeneration or preventing its degradation.^68^ In the present study, we found that synovial Fap levels positively correlated with OA progression and that genetic deletion or pharmacological inhibition of Fap significantly ameliorated OA symptoms. Furthermore, we showed that Fap degrades Col II both in vitro and in vivo, and this effect could be inhibited by both FAPi and its endogenous inhibitor Oln. Interestingly, Oln is expressed in the superficial layer of the articular cartilage and meniscus to form a protective barrier, which negatively correlates with OA progression. Genetic deletion of *Oln* promotes OA progression in a Fap-dependent manner, while intra-articular administration of rOln ameliorates OA. Together, this study identified Fap as a potential drug target to treat OA.

Fap is structurally and functionally similar to Dpp4,^69^ which is a dipeptidyl peptidase that shares many substrates with Fap.^70^ DPP4 inhibitors, such as sitagliptin, are orally available drugs that have been approved by the FDA to treat type 2 diabetes.^71^ Although the FAPi we used in this study is only 9-fold selective over Dpp4,^55^ it showed no therapeutic effects in Fap KO mice (Fig. 2). This finding suggests that Fap, but not Dpp4, is the downstream target of FAPi. Fap has both membrane-bound and soluble forms.^39^ Since synoviocytes do not infiltrate the cartilage matrix in OA,^9^ we reason that the soluble form of Fap secreted from OA synovium contributes to Col II degradation, which was confirmed by our ELISA analysis (Supplementary Fig. 6b). In contrast to OA, RA is characterized by severe inflammation and synoviocyte infiltration.^9^ Given that Fap^+^ synovial fibroblasts are known to play key roles in RA^72^ and that genetic deletion of *Fap* significantly ameliorates articular cartilage damage in a mouse model of RA,^50^ it is intriguing to test whether Fap inhibitors could also be used to treat RA in the future.

Previous studies showed that Fap degrades denatured Col I^35,63^ as well as MMP1-cleaved native Col I.^64,65^ Here, we provide evidence that Fap also degrades denatured Col II and MMP13-cleaved native Col II in vitro and primary chondrocyte-derived Col II ex vivo, thereby adding a new member to the substrate list of Fap. While MMP13 can cleave native Col II into large fragments, Fap helps to digest them into even smaller peptides (Fig. 4f). It is worth noting that Fap is a prolyl-specific serine protease and that 17% of COL2A1 amino acids are composed of proline. Thus, MMP13 and Fap work together for the efficient degradation of native Col II, the major component of the articular cartilage. Col II is also an in vivo substrate of Fap during OA progression. The Col II level was significantly increased after genetic deletion or inhibition of Fap (Figs. 2h and 3h) and significantly decreased after intra-articular administration of rFap (Fig. 5h). Unfortunately, we were unable to test whether FAPi attenuates OA progression in *Col2a1*-deficient mice, which died perinatally due to chondrodysplasia.^73,74^ Therefore, we cannot rule out the possibility that other substrates might also mediate the therapeutic effects of Fap inhibition.

MMPs play critical roles in cartilage degradation during OA progression,^75^ while tissue inhibitors of metalloproteinases (TIMPs) protect against OA.^76^ Whether similar antagonistic mechanisms exist to maintain the homeostasis of the articular cartilage remains poorly understood. Our previous study showed that Oln interacts with Fap and functions as an endogenous inhibitor to promote bone mineralization.^48^ Here, we found that Oln also localizes to the articular surface, which forms a protective barrier to prevent Fap-dependent degradation of Col II. During OA progression, cartilage erosion first occurs at the articular surface due to mechanical wear,^77^ which leads to Oln disruption and further deterioration of the cartilage matrix. Notably, Oln KO mice did not exhibit spontaneous OA, possibly because the Fap level is low in the knee joint under steady state (Fig. 1). Unlike FAPi, rOln cannot directly inhibit the serine protease activity of Fap in vitro (data not shown). However, Oln overexpression partially inhibited immunoprecipitated Fap (Fig. 4e), suggesting that they could form an inhibitory complex in vivo together with other protein adaptors. This study falls short of dissecting the detailed molecular mechanism by which Fap is inhibited by Oln, which could be achieved by purification of the inhibitory complex coupled with crystallography/cryo-EM analysis. We also noted that rOln showed limited therapeutic effects in vivo when dissolved in PBS (data not shown). However, HA seemed to be a better vehicle, probably by immobilizing rOln on the articular surface. Future studies are needed to optimize the vehicle and route of administration for FAPi and rOln and to test whether they could ameliorate OA progression in larger animal models and clinical trials.

## Materials and methods

### Human samples

This study was carried out in compliance with the Helsinki Declaration. Synovium, articular cartilage and synovial fluids were collected from the knee joints of OA patients (Kellgren–Lawrence grade^78^ 3 or 4) during arthroplasty procedures. Cartilage samples were examined under the stereoscope. The smooth regions with no obvious lesions were separated as normal cartilage, while the rough regions were separated as lesioned cartilage. Control synovial tissues were obtained from patients with no radiographic cartilage changes undergoing exploratory arthroscopy. Detailed patient information is summarized in Table S1.

### Mice

Fap KO mice (#024288)^56^ were obtained from the Jackson Laboratory and maintained by crossing with C57BL/6J wild-type mice (#000664). Parts of exons 4 and 5 were replaced by a LacZ-neo cassette in this strain, which led to the genetic ablation of *Fap*. The generation of Oln KO mice was previously described.^53^ Wild-type C57BL/6J mice were used for in vivo administration of rOln and FAPi. Male mice at approximately 10 weeks of age were used to perform the DMM surgery.

### qPCR

Total RNA was extracted from human samples or mouse primary chondrocytes using TRIzol reagent (Invitrogen) and then reverse transcribed into cDNA using a 5X All-In-One MasterMix kit (ABM). qPCR was performed using iTaq Universal SYBR Green Supermix (Bio-Rad) on a CFX96 real-time system (Bio-Rad). The PCR primers used included hACTB (NM_001101.5): 5’-ATT GGC AAT GAG CGG TTC-3’ and 5’-GGA TGC CAC AGG ACT CCA-3’; hFAP (NM_001291807.3): 5’-TGG CGA TGA ACA ATA TCC TAG A-3’ and 5’-ATC CGA ACA ACG GGA TTC TT-3’; hOLN/CLEC11A (NM_002975.3): 5’-GAG AGG GAG GCC CTG ATG-3’ and 5’-AAC AGT TCC GGC AGG ATT C-3’; mActb (NM_007393.5): 5’-GCT CTT TTC CAG CCT TCC TT-3’ and 5’-CTT CTG CAT CCT GTC AGC AA-3’; mAcan (NM_007424.3): 5’-TGA AGC AGA AGG TCT GGA CA-3’ and 5’-CCA GAA GGA ATC CCA CTA ACA-3’; mCol2a1 (NM_031163.3): 5’-GTC CCC CTG GCC TTA GTG-3’ and 5’-CCA CCA GCC TTC TCG TCA-3’; mMmp3 (NM_010809.2): 5’-TGC AGC TCT ACT TTG TTC TTT GA-3’ and 5’-AGA GAT TTG CGC CAA AAG TG-3’; mOln (NM_009131.3): 5’-AGG TCC TGG GAG GGA GTG-3’ and 5’-GGG CCT CCT GGA GAT TCT T-3’.

### Western blot

Protein extracts from human samples or primary chondrocytes were boiled at 95 °C for 10 min in 1× loading buffer and then subjected to SDS‒PAGE. Proteins were transferred onto PVDF blotting membranes, blocked with 5% dry milk in TBST buffer, and probed with anti-FAP (R&D, AF3715), anti-Gapdh (Proteintech, 60004), anti-Acan (Millipore Sigma, AB1031), anti-Col2a1 (Abcam, AB34712), anti-MMP3 (Abcam, AB52915) or anti-OLN/CLEC11A (R&D, BAF1904) antibodies overnight at 4 °C. After three washes in TBST buffer, the membrane was probed with donkey anti-sheep IgG-HRP (R&D, HAF016), goat anti-rabbit IgG-HRP (Absin Bioscience, abs20002) or goat anti-mouse IgG-HRP (Absin Bioscience, abs20001) secondary antibodies for 1 h at room temperature and then washed three times in TBST buffer. Chemiluminescent signals were detected with Pierce ECL Western blotting substrates (Thermo, 32106) or a SuperSignal™ West Femto Substrate Trial Kit (Thermo, 34094).

### Cryosection and immunostaining

Human synovium, cartilage or mouse knee joint samples were fixed in 4% paraformaldehyde overnight at 4 °C. Mouse knee joints were decalcified in 10% EDTA (pH 7.4) for 5–7 days. All samples were dehydrated in 30% sucrose for 1 day at room temperature and sectioned at 10 μm using the CryoJane tape-transfer system (Leica). Sections were incubated in blocking buffer (PBS with 5% horse serum) for 1 h at room temperature and then stained with anti-FAP (R&D, AF3715, 1:500), anti-OLN/CLEC11A (R&D, BAF1904, 1:500), anti-Oln/Clec11a (R&D systems, AF3729, 1:500), anti-beta galactosidase (GeneTex, GTX77365, 1:500) or anti-Col2a1 (Boster, BA0533, 1:200) antibodies in blocking buffer overnight at 4 °C. Slides were washed 3 times with PBS and then stained with Alexa Fluor 647 donkey anti-Sheep IgG (H+L) (Thermo Fisher Scientific, A21448, 1:500), Alexa Fluor Plus 555 donkey anti-goat IgG (H+L) (Thermo Fisher Scientific, A32816, 1:500) or Alexa Fluor 488 donkey anti-chicken IgY (IgG) (H+L) (Jackson ImmunoResearch, 703-545-155, 1:500) secondary antibodies in blocking buffer for 1 h at room temperature. Slides were then washed 3 times with PBS, stained with 1 μg/ml DAPI for 1 min and mounted with Anti-fade Prolong Gold (Invitrogen). Images were acquired using an Olympus IX73 microscope.

### Genotyping

To genotype Fap KO mice, the following primers were used: 5’-TTT GGG CCA GGG TTT TCC CAG TCA C-3’, 5’-TGG ACA GGG AGG AAG ACA AG-3’ and 5’-GAG GGC AGA GGC TTA GTG TG-3’ (WT: 361 bp; Heterozygous: 230 and 361 bp; KO: 230 bp). To genotype Oln^+/+^, Oln^+/−^, and Oln^−/−^ mice, the following primers were used: 5’-TTT GGG TGC TGG GAA GCC C-3’ and 5’-TTG CAC TGA GTC GCG GGT G-3’ (Oln^+/+^: 910 bp; Oln^+/−^ or Oln^−/−^: 538 bp). To distinguish between Oln^+/−^ and Oln^−/−^ mice, the following primers were used: 5’-GAG GAA GAG GAA ATC ACC ACA GC-3’ and 5’-TTG CAC TGA GTC GCG GGT G-3’ (Oln^+/−^: 482 bp; Oln^−/−^: no amplification product).

### DMM model

Mice were anesthetized with 1.5% pentobarbital (80 mg·kg^−1^ body weight). Ketoprofen (5 mg·kg^−1^ body weight) was administered subcutaneously for pain relief. Knee joints were shaved and sterilized with Betadine and 70% ethanol, and a 4 mm longitudinal incision was made from the inferior pole of the patellar to the proximal tibial plateau. The joint capsule was opened medial to the patellar tendon with microiris scissors. The fat pad over the intercondylar area was bluntly dissected. For the DMM operation, the medial meniscotibial ligament was incised with microiris scissors, and the mobility of the anterior horn of the medial meniscus was tested. For the sham operation, no other procedure was performed. The joint capsule and the skin were then closed separately with a 7–0 cutting PGA suture line (Jinhuan Medical).

### Intra-articular injection

Mice were randomly chosen for each intervention group. Mice were anesthetized with isoflurane, and knee joints were sterilized with 70% ethanol. The needle of the insulin syringe was sagittally inserted into the intercondylar area of the mouse knee, where 10 μl of FAPi (MCE, HY-101801), rFap (R&D Systems, 8647-SE-010) or rOln was injected. For the prevention model, intra-articular injections of FAPi (40 μg·kg^−1^ body weight, in PBS), rOln (240 μg·kg^−1^ body weight, in 0.05 mg·mL^−1^ HA) or the corresponding vehicle were given 3 days after the surgery. For the treatment model, intra-articular injections of FAPi, rFap (40 μg·kg^−1^ body weight, in PBS), rOln or the corresponding vehicle were given 4 weeks after DMM surgery.

### Histomorphometry analysis

Dissected mouse knee joints were fixed in 4% paraformaldehyde overnight at 4 °C and decalcified in 10% EDTA for 14 days. Paraffin-embedded knee joints were sectioned at 6-μm thickness and stained with hematoxylin, 0.5% Safranin O solution and 0.2% Fast Green solution. Grading was performed by calculating OARSI scores (medial tibial plateau)^79^ and synovitis scores (summation of lining layer enlargement, density of resident cells, and inflammatory infiltration).^80^ Subchondral bone thickness was measured as the mean distance of five evenly distributed measuring points between the lower edge of the articular cartilage and the roof of the cancellous bone.^81,82^ The average score of 6 knee sections was calculated for statistical analysis.

### Micro-CT analysis

Dissected mouse knee joints were fixed in 4% paraformaldehyde overnight at 4 °C and replaced with PBS before micro-CT analysis. Knee joints were scanned at an isotropic voxel size of 7 μm, with a peak tube voltage of 70 kV and current of 0.114 mA (mCT 35; Scanco Medical AG, Bassersdorf, Switzerland). A three-dimensional Gaussian filter (*s* = 0.8) with a limited, finite filter support of one was used to suppress noise in the images, and a threshold of 220–1 000 was used to segment mineralized bone from the air and soft tissues. The region of interest was selected for calcified osteophyte tissues, and the bone volume was calculated to measure the osteophyte size.

### Primary cell culture

Primary chondrocyte culture was performed as previously described.^57^ Briefly, P5-P7 newborn mice were sacrificed, and cartilage from the knee and shoulder joints was dissected with ophthalmic scissors and micro forceps. The cartilage tissues were rinsed three times with PBS and cut into smaller pieces. Cartilage pieces were incubated in digestion buffer (DMEM, 3 mg/ml collagenase type II, 4 mg·mL^−1^ Dispase II, 1% penicillin/streptomycin) twice at 37 °C for 45 min each and incubated in diluted digestion buffer (6×) overnight at 37 °C. Dissociated chondrocytes were then filtered, centrifuged, and seeded in 24-well plates at 2 × 10^5^ cells per well. Chondrocytes were cultured with DMEM (Corning, 10-014-CV) plus 10% fetal bovine serum (GIBCO, 10270-106), 2 mmol·L^−1^ GlutaMAX Supplement (Thermo Fisher Scientific, 35050061) and 1% penicillin/streptomycin. The culture medium was changed after 8–10 h. All experiments were performed within 3 days after seeding. For human synovial fibroblast cultures, synovial tissues (from 1 male and 2 females, average age: 52.33) were rinsed three times with DPBS (1% penicillin/streptomycin) and minced with scissors to smaller pieces. Tissues were then incubated in digestion buffer (DMEM, 1 mg·mL^−1^ collagenase type I, 1% penicillin/streptomycin) at 37 °C for 45 min and vortexed every 3–5 min. Dissociated synovial fibroblasts were filtered, centrifuged, seeded in 10 cm dishes, and cultured with DMEM (Corning, 10-014-CV) plus 10% fetal bovine serum (GIBCO, 10270-106) and 1% penicillin/streptomycin. The culture medium was changed after 8–10 h. For IL-1β stimulation experiments, synovial fibroblasts were passaged in 48-well plates at 5 × 10^4^ cells per well and stimulated with 10 ng·mL^−1^ IL-1β for different durations.

### Enzymatic digestion

The enzymatic reaction was initiated by adding different doses of rFap (R&D Systems, 8647-SE-010), native or denatured (heated at 95 °C for 10 min) bovine Col II (Chondrex, 20021) into 10 μL PBS buffer and incubated for 1–24 h at 37 °C. For the FAPi inhibition experiment, FAPi and rFap were preincubated for 30 min before denatured Col II was added to the reaction mixture. For the MMP13 predigestion experiment, rMMP13 and native Col II were incubated in assay buffer (50 mmol·L^−1^ Tris, 1 mmol·L^−1^ CaCl_2_, 150 mmol·L^−1^ NaCl, 0.05% Brij-35) at 37 °C for 12 h, after which EDTA (1 mmol·L^−1^ final concentration) and rFap were added to the reaction mixture for another 12 h. Different doses of rFap and 10 μg·mL^−1^ native or denatured (heated at 95 °C for 10 min) recombinant human Aggrecan (G1-IGD-G2 domain, R&D Systems, 1220-PG-025) were incubated in 10 μL PBS buffer at 37 °C for 24 h. Enzymatic reactions were stopped by adding loading buffer (NCM, WB2001) and boiling at 95 °C for 10 min. Then, the samples were subjected to 6% SDS‒PAGE. Gels were stained with a Colloidal Blue Staining Kit (Thermo, LC6025). Grayscales were calculated with ImageJ.

### Immunoprecipitation

Fap-HA, Oln-Flag, and control GFP constructs were transfected alone or together into the HEK293T cell line (ATCC) using Lipofectamine 3000 (Thermo Fisher Scientific). Three days after transfection, cells were washed with ice-cold PBS and lysed in 500 μL ice-cold RIPA buffer (100 mmol·L^−1^ Tris-HCl, 150 mmol·L^−1^ NaCl, 1% Triton-X-100, 1X protease inhibitor cocktails). After centrifugation at 12 000 r·min^−1^ for 5 min, the supernatants were incubated with 10 μL anti-HA affinity gel (Sigma) overnight at 4 °C and washed three times with RIPA buffer and three times with PBS. Immunoprecipitated proteins were incubated with denatured Col II at 37 °C for 24 h and then subjected to 6% SDS‒PAGE and colloidal blue staining as described above. Immunoprecipitation efficiency was detected by western blotting with anti-HA (Sigma, H6908) and anti-Flag (Sigma, F1804) antibodies.

### ELISA

Secreted FAP levels in human synovial fluids were measured using a Human FAP ELISA Kit (Abcam, ab193701) according to the manufacturer’s instructions. Briefly, human synovial fluids were diluted 1:100 using PBS buffer, and 100 μL of diluted synovial fluid was added to a precoated 96-well ELISA plate at 4 °C for 16 h. The plate was then washed 4 times with 1× wash buffer, and 100 μL of biotinylated human FAP detection antibody (diluted in 1× assay diluent B) was added and incubated at room temperature for 1 h. After washing with 1× wash buffer 4 times, 100 μL of HRP Streptavidin solution (diluted in 1× assay diluent B) was added and incubated at room temperature for 45 min. After washing with 1× wash buffer 4 times, 100 μL of TMB one-step substrate reagent was added to each well and incubated at room temperature in the dark for 30 min. Finally, 50 μL of stop solution was added to each well, and the optical density was measured at 450 nm.

### rOln purification

rOln purification has been previously described.^53^ Briefly, HEK293 cells (ATCC) stably overexpressing mouse Oln-Flag were lysed and immunoprecipitated with anti-Flag M2 affinity gel (Sigma) overnight at 4 °C. The beads were washed three times with RIPA buffer and eluted using 100 μg·mL^−1^ 3× FLAG peptide in elution buffer (50 mmol·L^−1^ HEPES, 150 mmol·L^−1^ NaCl and 10% glycerol, pH = 7.5). Eluted protein was concentrated by Amicon Ultra15 Centrifugal Filter Units (Millipore) and quantified by SDS‒PAGE with rOln standards (R&D Systems, 1904-SC-025/CF).

### Statistics

The statistical significance of differences between two groups was assessed using two-tailed Student’s *t* tests. The statistical significance of differences among more than two groups was assessed using one-way ANOVAs with Tukey’s multiple comparison tests or two-way ANOVAs with Sidak’s multiple comparison tests. All data are presented as the mean ± SD. *P* values less than 0.05 were considered significant (*P < 0.05, **P < 0.01, ***P < 0.001).

## Supplementary information

Supplementary figures

Supplementary figures and legends

## Data Availability

All data are available from the corresponding authors upon reasonable request.
