# Supplementary material for: Inhibition of fibroblast activation protein ameliorates cartilage matrix degradation and osteoarthritis progression
Source: Bone Res. 2023 Jan 2;11:3. doi: 10.1038/s41413-022-00243-8 (PMC9806108; doi:10.1038/s41413-022-00243-8)

Supplementary Figure 1

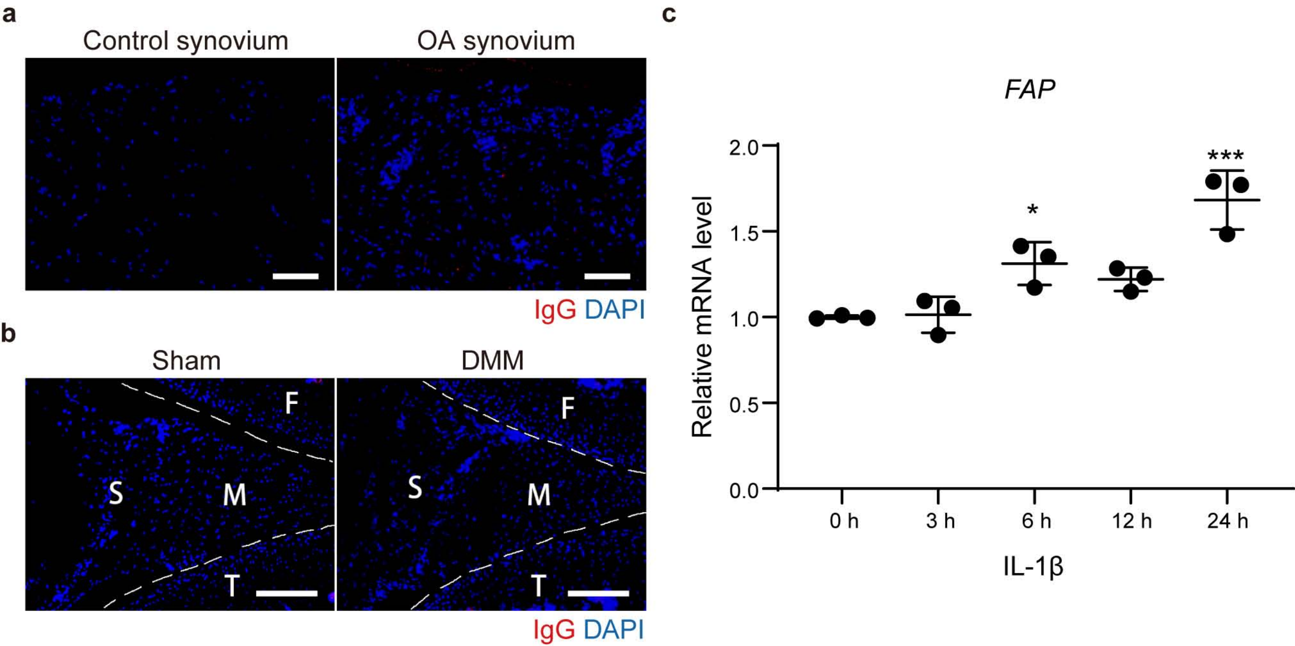

Supplementary Figure 2

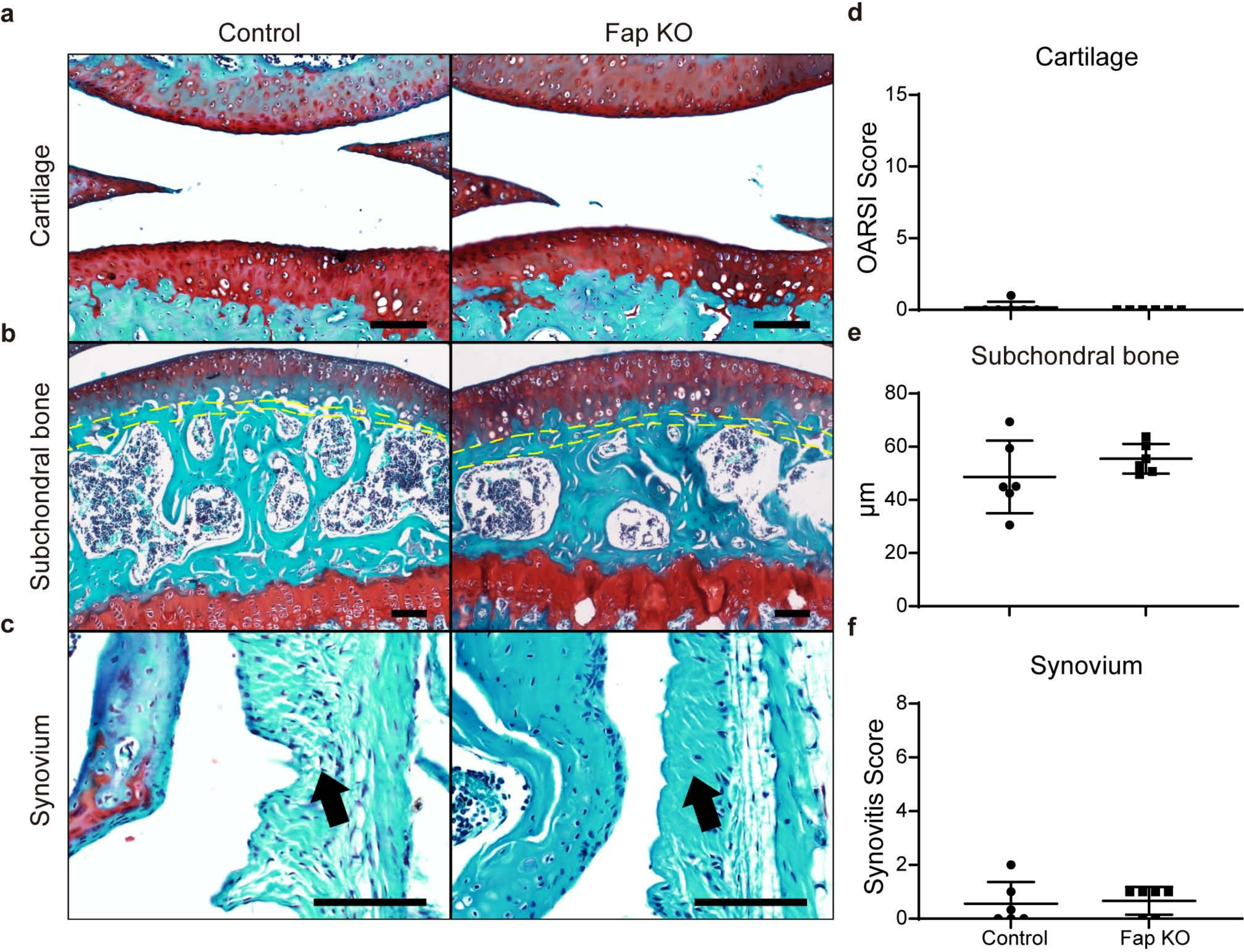

Supplementary Figure 3

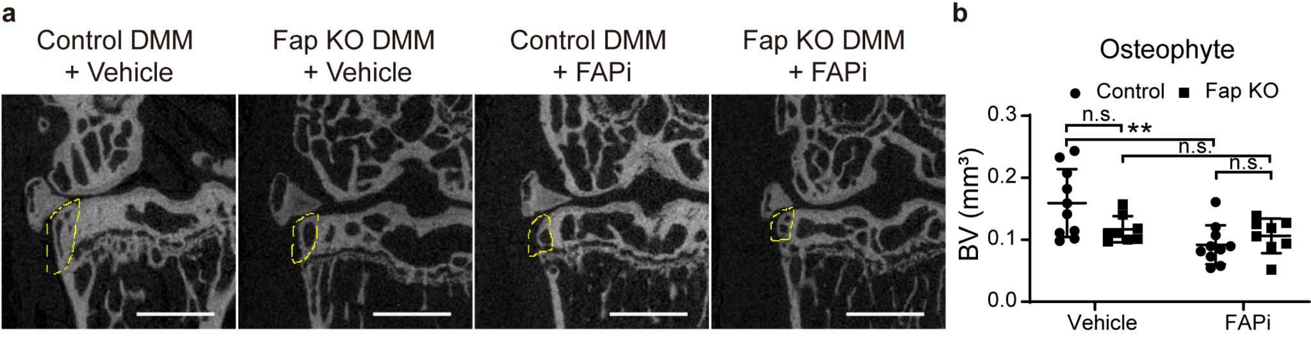

Supplementary Figure 4

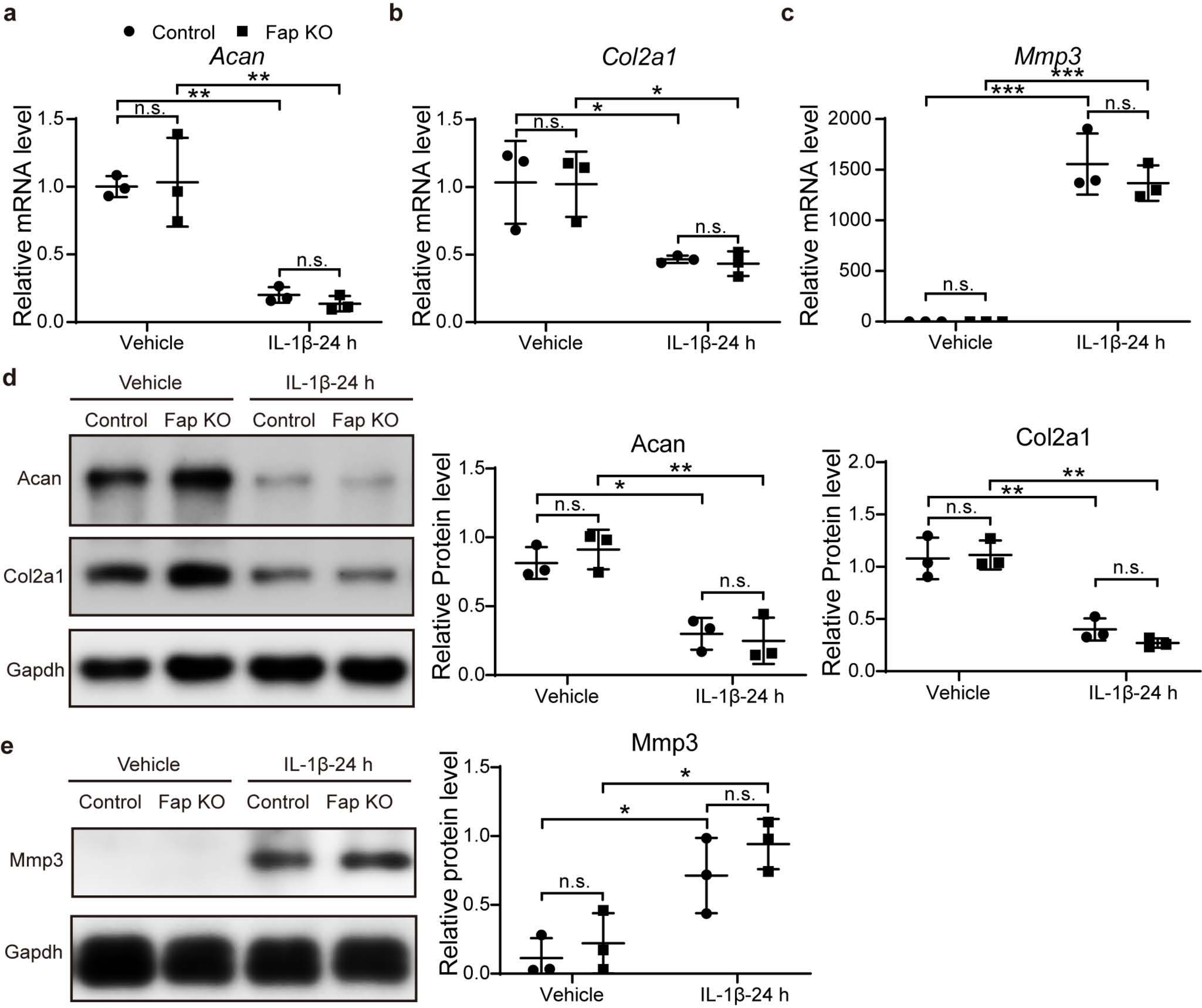

Supplementary Figure 5

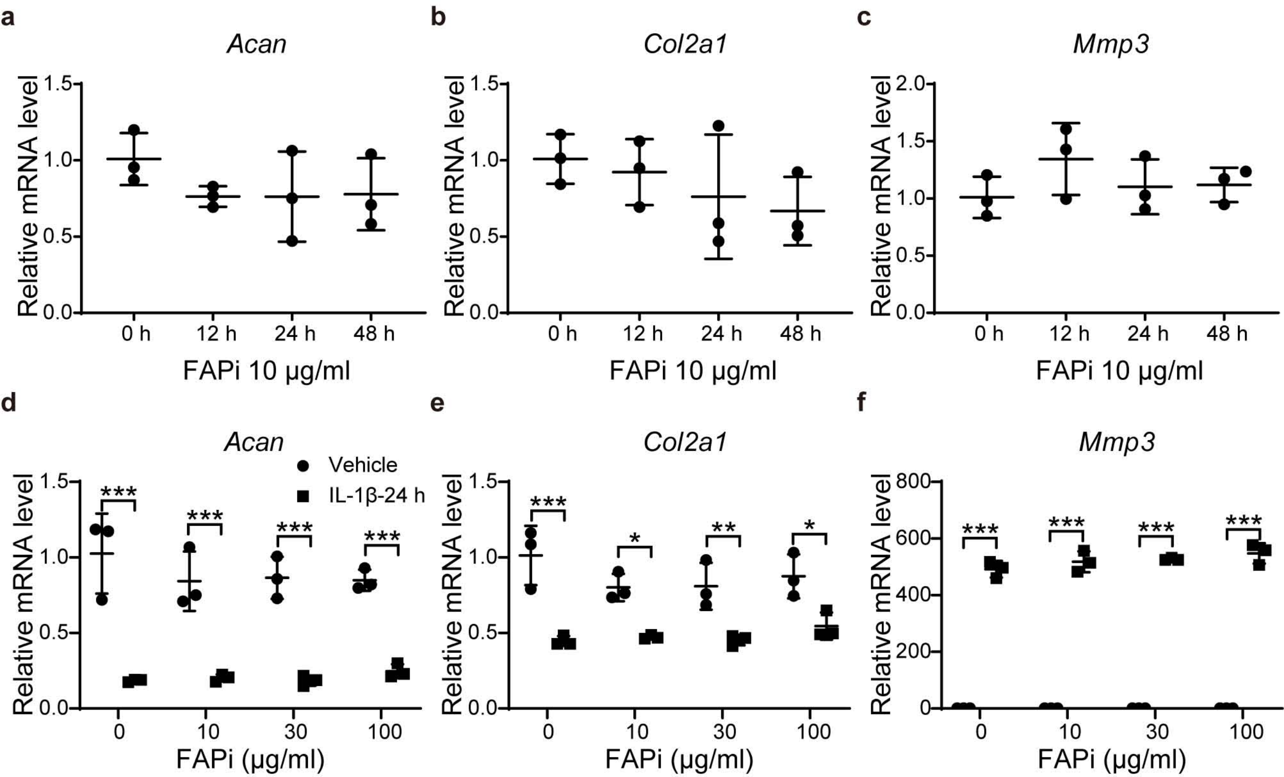

Supplementary Figure 6

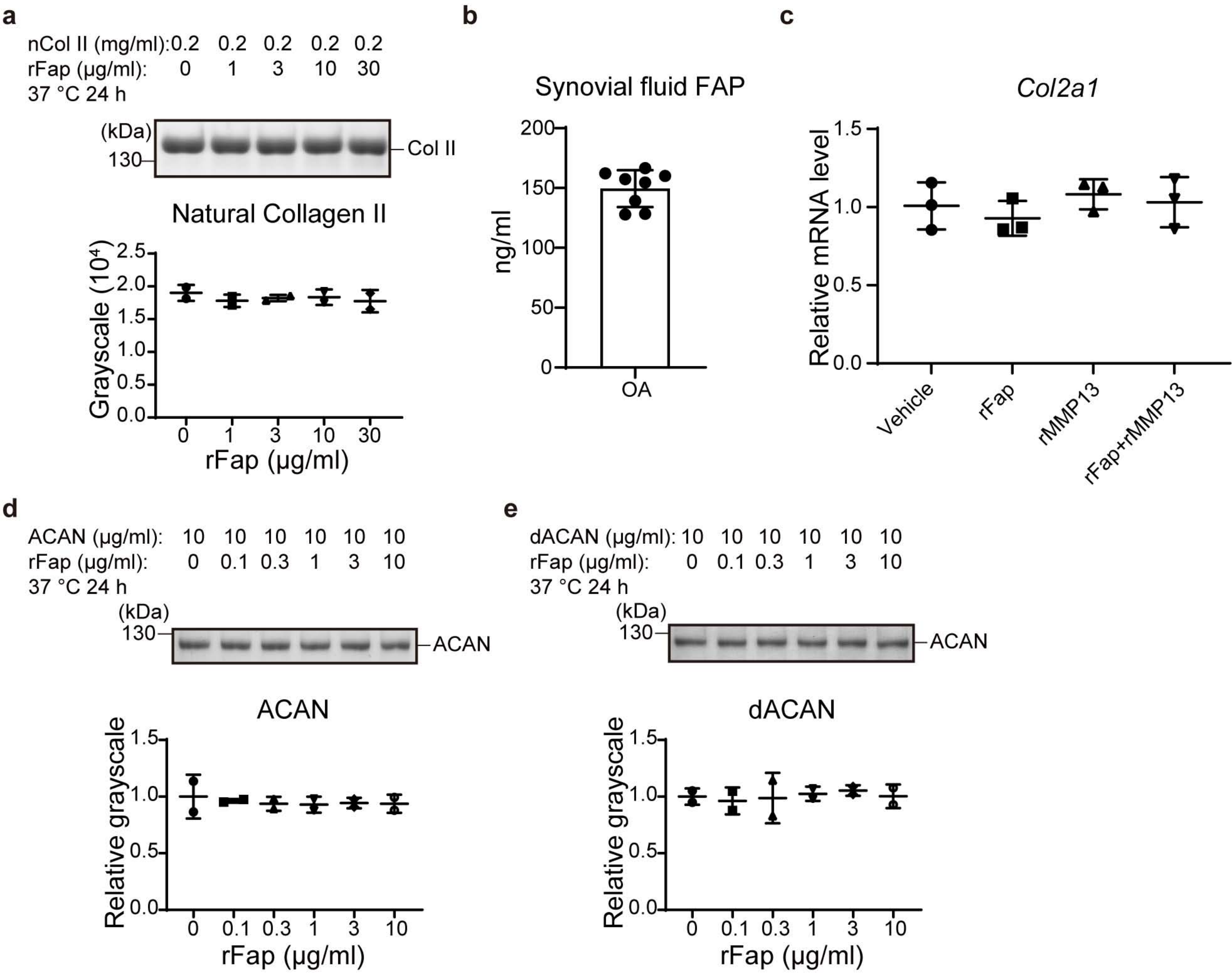

Supplementary Figure 7

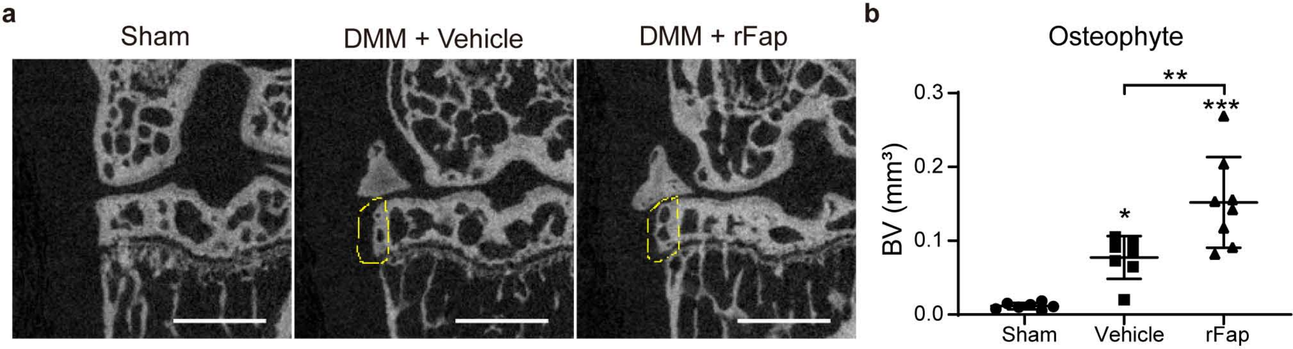

Supplementary Figure 8

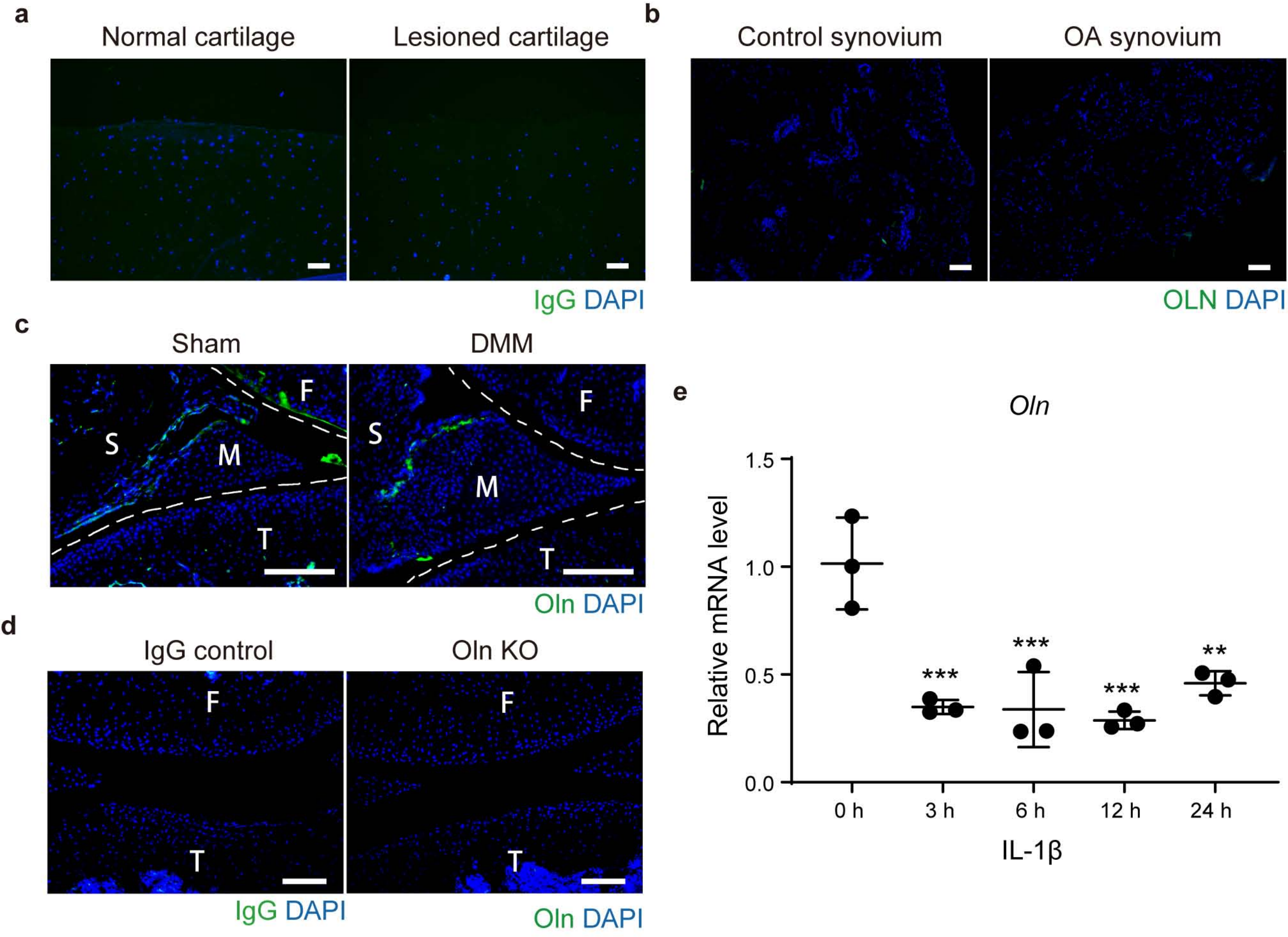

Supplementary Figure 9

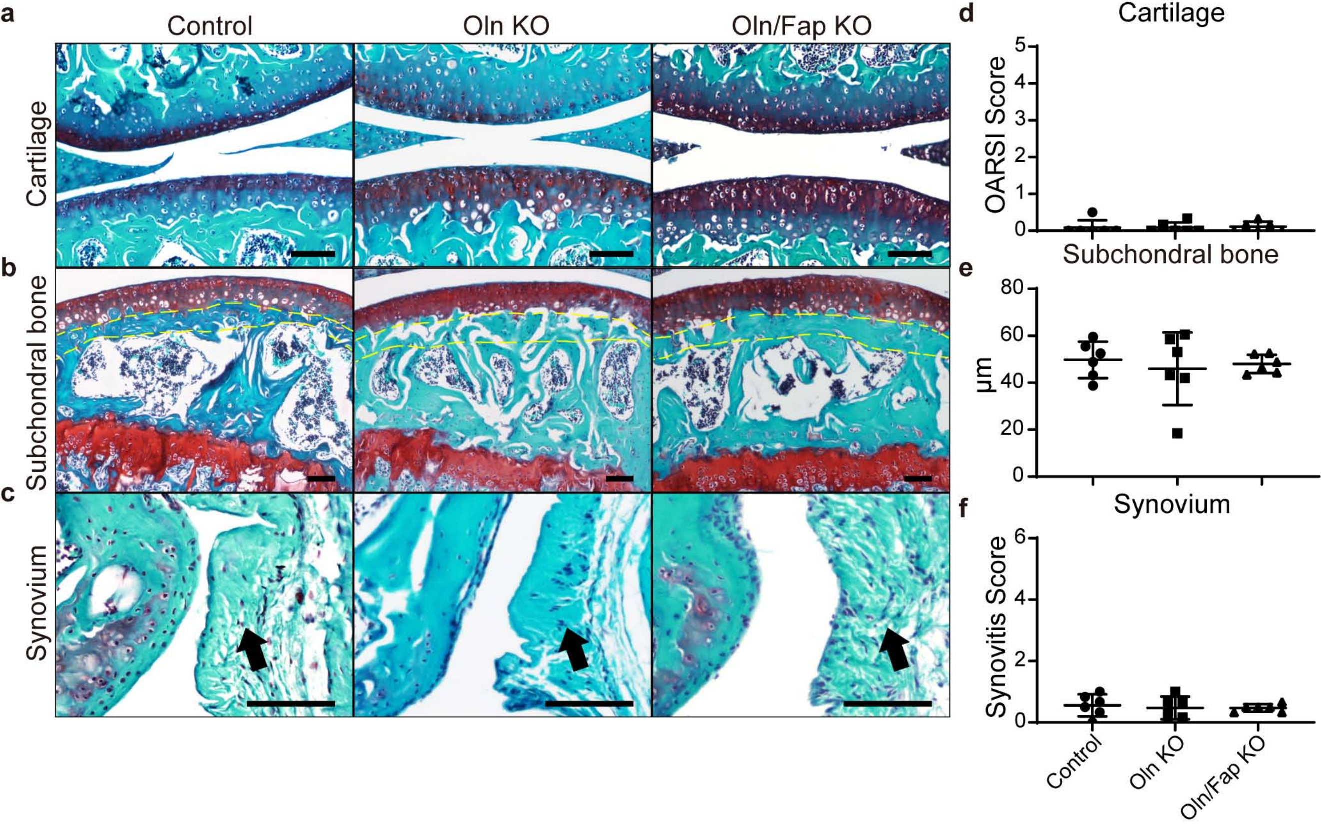

Supplementary Figure 10

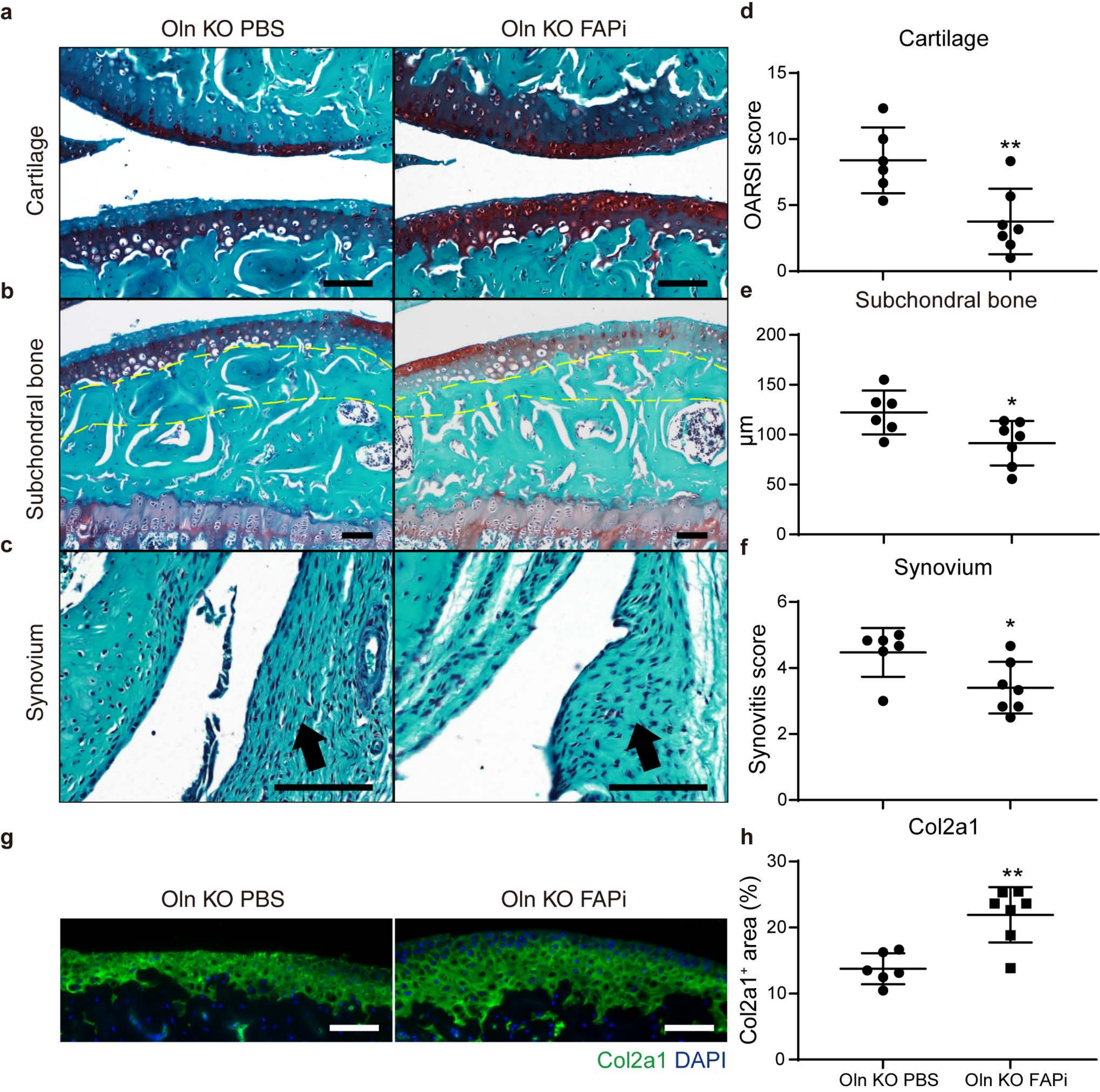

Supplementary Figure 11

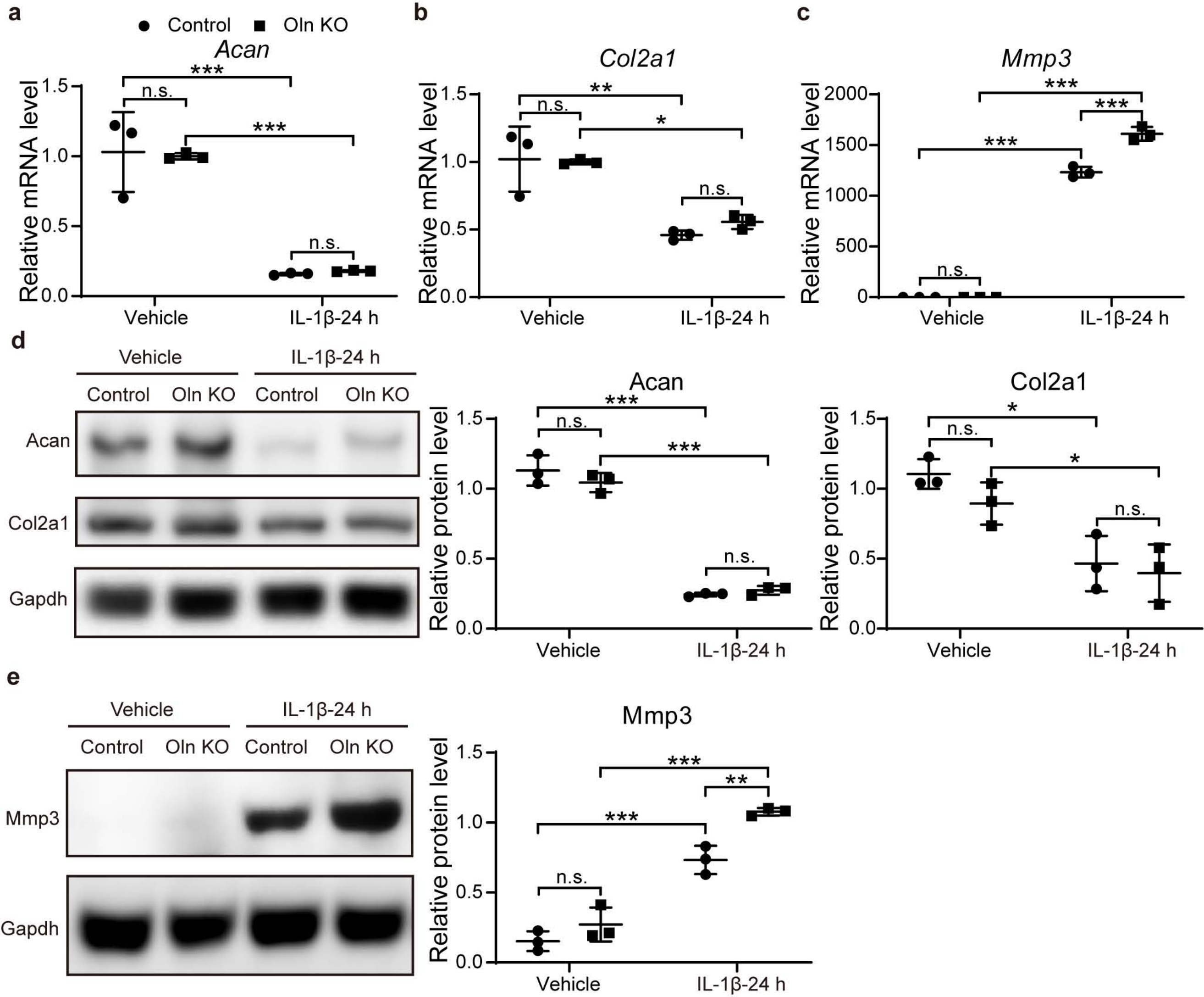

Supplementary Figure 12

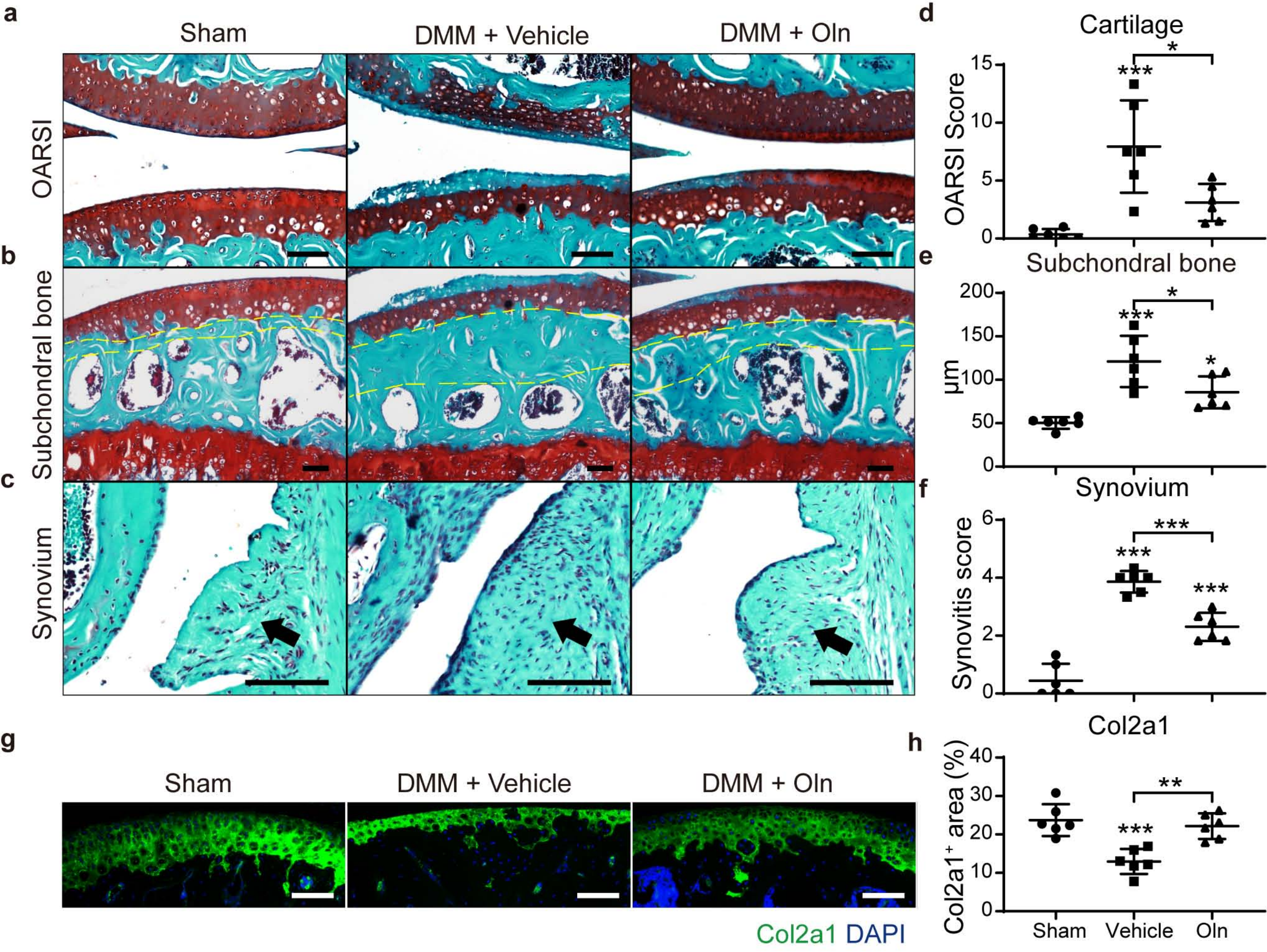

Supplement: Supplementary file 1 — Supplementary figures [file 41413_2022_243_MOESM1_ESM.pdf]
