# Supplementary material for: Inhibition of fibroblast activation protein ameliorates cartilage matrix degradation and osteoarthritis progression
Source: Bone Res. 2023 Jan 2;11:3. doi: 10.1038/s41413-022-00243-8 (PMC9806108; doi:10.1038/s41413-022-00243-8)
Supplement: Supplementary file 2 — Supplementary figures and legends [file 41413_2022_243_MOESM2_ESM.docx]

**SUPPLEMENTARY FIGURES AND LEGENDS**

**Supplementary Figure 1. Negative controls for the immunostaining of human and mouse Fap**

(**a**) Immunostaining of isotype control (IgG) in the synovium of control and OA patients. DAPI staining indicates the nucleus. Scale bars: 100 μm.

(**b**) Immunostaining of isotype control (IgG) in the knee joint of sham and DMM-treated mice. Sham or DMM surgery was performed in 8-week-old Fap^LacZ/+^ mice, which were sacrificed 8 weeks later (F: Femur; T: Tibia; M: Meniscus; S: Synovium). DAPI staining indicates the nucleus. Scale bars: 100 μm.

(**c**) qPCR analysis of *FAP* expression in IL-1β-stimulated primary human synovial fibroblasts (n = 3 independent experiments).

The statistical significance was assessed using one-way ANOVAs with Tukey’s multiple comparison tests (**P* <0.05, ****P* < 0.001).

**Supplementary Figure 2. Genetic ablation of *Fap* does not affect knee joint morphology at 4 months of age**

(**a** to **c**) Safranin O/Fast Green staining in the contralateral knee joints without DMM surgery. Representative images of articular cartilage (**a**, top: femur, bottom: tibia), subchondral bone plate (**b**) and synovium (**c**) in control and Fap KO mice are shown (*n* = 6 mice per genotype). Arrows indicate the synovium. Scale bars: 100 μm.

(**d** to **f**) Quantification of the OARSI score (**d**), subchondral bone thickness (**e**) and synovitis score (**f**).

The statistical significance was assessed using two-tailed Student’s unpaired t tests.

**Supplementary Figure 3. Micro-CT analysis of osteophytes after Fap inhibition**

(**a** and **b**) Micro-CT analysis of control and Fap KO mice treated with FAPi or vehicle after DMM surgery (**a**) with quantification (**b**). Weekly intra-articular administration of FAPi (40 μg/kg body weight) or vehicle (PBS) was started 3 days after DMM surgery in 10-week-old mice and continued for 8 weeks before micro-CT analysis (*n* = 8-10 mice per genotype in each treatment group). Yellow dotted lines indicate the osteophytes. Scale bars: 1 mm.

The statistical significance was assessed using two-way ANOVAs with Sidak’s multiple comparison tests (*P <0.05, **P < 0.01, ***P < 0.001).

**Supplementary Figure 4. Genetic ablation of Fap does not affect chondrocyte functions ex vivo**

(**a** to **c**) qPCR analysis of mouse *Acan* (**a**), *Col2a1* (**b**) and *Mmp3* (**c**) mRNA levels in primary chondrocytes of control and Fap KO mice (*n* = 3 independent experiments). Chondrocytes were stimulated with or without 10 ng/ml IL-1β for 24 h.

(**d** and **e**) Western blot analysis of Acan, Col2a1 (**d**) and MMP3 (**e**) protein levels in primary chondrocytes of control and Fap KO mice. Chondrocytes were stimulated with or without 10 ng/ml IL-1β for 24 h (*n* = 3 independent experiments).

The statistical significance was assessed using two-way ANOVAs with Sidak’s multiple comparison tests (**P* <0.05, ***P* < 0.01, ****P* < 0.001).

**Supplementary Figure 5. FAPi does not affect chondrocyte functions *ex vivo***

(**a** to **c**) qPCR analysis of mouse *Acan* (**a**), *Col2a1* (**b**) and *Mmp3* (**c**) mRNA levels in primary chondrocytes of wild-type mice. Chondrocytes were stimulated with 10 μg/ml FAPi for 0-48 h (*n* = 3 independent experiments).

(**d** to **f**) qPCR analysis of mouse *Acan* (**d**), *Col2a1* (**e**) and *Mmp3* (**f**) mRNA levels in primary chondrocytes of wild-type mice (*n* = 3 mice per group). Chondrocytes were stimulated with or without 10 ng/ml IL-1β plus 0-100 μg/ml FAPi for 24 h.

The statistical significance was assessed using one-way ANOVAs with Tukey’s multiple comparison tests (**P* <0.05, ***P* < 0.01, ****P* < 0.001).

**Supplementary Figure 6. rFap cannot degrade native Col II or Acan *in vitro***

(**a**) Fap cannot degrade native Col II. Native Col II were incubated with different amounts of rFap at 37 °C for 24 h. Samples were separated by SDS‒PAGE and quantified by colloidal blue staining (*n* = 2 independent experiments).

(**b**) ELISA measurement of human FAP levels in synovial fluids of OA patients (*n* = 8 patients).

(**c**) qPCR analysis of mouse *Col2a1* mRNA levels in primary chondrocytes (*n* = 3 independent experiments). Chondrocytes were stimulated with vehicle control (PBS), 200 ng/ml rFap, 200 ng/ml rMMP13, or 200 ng/ml rFap plus 200 ng/ml rMMP13 for 24 h.

(**d** and **e**) Fap cannot degrade native (**d**) or denatured (**e**) Acan. Native (**d**) or denatured (**e**) Acan (G1-IGD-G2) were incubated with different amounts of rFap at 37 °C for 24 h. Samples were separated by SDS‒PAGE and quantified by colloidal blue staining (*n* = 2 independent experiments).

**Supplementary Figure 7. Micro-CT analysis of osteophytes after rFap injection**

(**a** and **b**) Micro-CT analysis of wild-type mice treated with rFap or vehicle after DMM surgery (**a**) with quantification (**b**). Weekly intra-articular administration of rFap (40 μg/kg body weight) or vehicle (PBS) was performed 4 weeks after DMM surgery in 10-week-old mice and continued for 8 weeks before micro-CT analysis (*n* = 7-8 mice per genotype in each treatment group). Yellow dotted lines indicate the osteophytes. Scale bars: 1 mm.

The statistical significance was assessed using one-way ANOVAs with Tukey’s multiple comparison tests (*P <0.05, **P < 0.01, ***P < 0.001).

**Supplementary Figure 8. Negative controls for the immunostaining of Oln**

(**a**) Immunostaining of isotype control (IgG) in normal and lesioned articular cartilage of OA patients. DAPI staining indicates the nucleus. Scale bars: 100 μm.

(**b**) Immunostaining of human OLN in the synovium of control and OA patients. DAPI staining indicates the nucleus. Scale bars: 100 μm.

(**c**) Immunostaining of mouse Oln in the knee joints of sham and DMM-treated mice. Sham and DMM surgeries were performed in 8-week-old mice, which were sacrificed 8 weeks later. Immunostaining of mouse Oln was performed at the posterior angle of the medial meniscus (F: Femur; T: Tibia; M: Meniscus; S: Synovium). DAPI staining indicates the nucleus. Scale bars: 100 μm.

(**d**) Immunostaining of isotype control (IgG, left) and mouse Oln (right) in the knee joints of sham-operated (left) and Oln KO (right) mice (F: Femur; T: Tibia). DAPI staining indicates the nucleus. Scale bars: 100 μm.

(**e**) qPCR analysis of mouse *Oln* mRNA levels in primary chondrocytes. Chondrocytes were stimulated with 10 ng/ml IL-1β for different durations (n = 3 independent experiments).

The statistical significance was assessed using one-way ANOVAs with Tukey’s multiple comparison tests (***P* < 0.01, ****P* < 0.001).

**Supplementary Figure 9. Genetic ablation of *Oln* does not affect knee joint morphology at 4 months of age**

(**a** to **c**) Safranin O/Fast Green staining in the contralateral knee joints without DMM surgery. Representative images of articular cartilage (**a**, top: femur, bottom: tibia), subchondral bone plate (**b**) and synovium (**c**) in control, Oln KO and double KO mice are shown (*n* = 6 mice per genotype). Arrows indicate the synovium. Scale bars: 100 μm.

(**d** to **f**) Quantification of the OARSI score (**d**), subchondral bone thickness (**e**) and synovitis score (**f**).

The statistical significance was assessed using one-way ANOVAs with Tukey’s multiple comparison tests. Data are presented as the mean ± SD.

**Supplementary Figure 10. Pharmacological inhibition of Fap ameliorates joint symptoms in Oln KO mice**

(**a** to **c**) Safranin O/Fast Green staining in Oln KO mice treated with FAPi or vehicle after DMM surgery. Weekly intra-articular administration of FAPi (40 μg/kg body weight) or vehicle (PBS) was performed 3 days after DMM surgery in 10-week-old mice and continued for 8 weeks before paraffin sectioning and safranin O/fast green staining of the knee joints. Representative cartilage erosion (**a**, top: femur, bottom: tibia), subchondral bone thickening (**b**) and synovitis (**c**) images are shown (*n* = 6-7 mice per group). Yellow dotted lines indicate the subchondral bone plate. Arrows indicate the synovium. Scale bars: 100 μm.

(**d** to **f**) Quantification of the OARSI score (**d**), subchondral bone thickness (**e**) and synovitis score (**f**).

(**g** and **h**) Immunostaining of Col II in the knee joints (**g**) with quantification (**h**) (*n* = 6-7 mice per group). DAPI staining indicates the nucleus. Scale bars: 100 μm.

The statistical significance was assessed using two-tailed Student’s unpaired t tests. Data are presented as the mean ± SD (**P* <0.05, ***P* < 0.01).

**Supplementary Figure 11. Genetic ablation of *Oln* elevates Mmp3 levels in chondrocytes upon IL-1β stimulation**

(**a** to **c**) qPCR analysis of mouse *Acan* (**a**), *Col2a1* (**b**) and *Mmp3* (**c**) mRNA levels in primary chondrocytes of control and Oln KO mice. Chondrocytes were stimulated with 10 ng/ml IL-1β for 24 h (*n* = 3 independent experiments).

(**d** and **e**) Western blot analysis of Acan, Col2a1 (**d**) and MMP3 (**e**) protein levels in primary chondrocytes of control and Oln KO mice. Chondrocytes were stimulated with or without 10 ng/ml IL-1β for 24 h (*n* = 3 independent experiments).

The statistical significance was assessed using two-way ANOVAs with Sidak’s multiple comparison tests. Data are presented as the mean ± SD (**P* <0.05, ***P* < 0.01, ****P* < 0.001).

**Supplementary Figure 12. rOln prevents OA progression in the DMM model**

(**a** to **c**) Safranin O/Fast Green staining in wild-type mice treated with rOln or vehicle after DMM surgery. Weekly intra-articular administration of rOln (240 μg/kg body weight) or vehicle (0.05 mg/ml hyaluronic acid) was performed 3 days after DMM surgery in 10-week-old mice and continued for 8 weeks before paraffin sectioning and safranin O/fast green staining of the knee joints. Representative cartilage erosion (**a**, top: femur, bottom: tibia), subchondral bone thickening (**b**) and synovitis (**c**) images are shown (*n* = 6 mice per treatment). Yellow dotted lines indicate the subchondral bone plate. Arrows indicate the synovium. Scale bars: 100 μm.

(**d** to **f**) Quantification of the OARSI score (**d**), subchondral bone thickness (**e**) and synovitis score (**f**).

(**g** and **h**) Immunostaining of Col II in the knee joints (**g**) with quantification (**h**). DAPI staining indicates the nucleus (*n* = 6 mice per treatment). Scale bars: 100 μm.

The statistical significance was assessed using one-way ANOVAs with Tukey’s multiple comparison tests. Data are presented as the mean ± SD (**P* <0.05, ***P* < 0.01, ****P* < 0.001).

**Supplementary Table 1. Detailed patient information.**

| No | Group | Age/Sex | KL grade | Height (m) | Weight (kg) | BMI (kg/m2) | Other disease | | | | Samples | Use | | | |
| --- | --- | --- | --- | --- | --- | --- | --- | --- | --- | --- | --- | --- | --- | --- | --- |
|  |  |  |  |  |  |  | RA | DM | HT | CA |  | qPCR | WB | ELISA | IF |
| 1 | OA | 78/M | 4 | 1.72 | 80 | 27.04 | - | - | + | - | Cartilage, synovium & synovial fluid | √ |  | √ |  |
| 2 |  | 70/F | 4 | 1.57 | 65 | 26.37 | - | - | + | - |  | √ |  | √ | √ |
| 3 |  | 61/F | 4 | 1.68 | 60 | 21.26 | - | - | - | - |  | √ |  | √ | √ |
| 4 |  | 64/F | 3 | 1.58 | 59 | 23.63 | - | - | - | - |  | √ |  | √ |  |
| 5 |  | 49/F | 3 | 1.55 | 69 | 28.72 | - | + | + | - |  | √ |  | √ |  |
| 6 |  | 73/F | 4 | 1.55 | 64.5 | 26.85 | - | + | + | - |  | √ | √ | √ |  |
| 7 |  | 70/M | 4 | 1.74 | 75 | 24.77 | - | - | + | - |  | √ | √ | √ |  |
| 8 |  | 67/F | 4 | 1.58 | 62 | 24.84 | - | - | - | - |  | √ | √ | √ |  |
| 9 | Control | 53/F | 0 | 1.61 | 57 | 21.99 | - | - | - | - | Cartilage & synovium | √ |  |  |  |
| 10 |  | 50/F | 0 | 1.68 | 62 | 21.97 | - | - | - | - |  | √ |  |  | √ |
| 11 |  | 35/M | 0 | 1.79 | 95 | 29.65 | - | - | - | - |  | √ |  |  |  |
| 12 |  | 47/M | 0 | 1.76 | 79 | 25.50 | - | - | - | - |  | √ | √ |  |  |
| 13 |  | 55/F | 0 | 1.53 | 60 | 25.63 | - | - | - | - |  | √ | √ |  |  |
| 14 |  | 58/F | 0 | 1.66 | 70 | 25.40 | - | - | + | - |  | √ | √ |  |  |
| 15 |  | 42/F | 0 | 1.65 | 73 | 26.81 | - | - | - | - |  | √ |  |  | √ |
| 16 |  | 66/F | 1 | 1.58 | 55 | 22.03 | - | + | - | - |  | √ |  |  |  |

RA, rheumatoid arthritis; DM, diabetes mellitus; HT, hypertension; CA, cancer; +, present; -, absent
